# *CRNDE* acts as an epigenetic modulator of the p300/YY1 complex to promote HCC progression and therapeutic resistance

**DOI:** 10.1186/s13148-022-01326-3

**Published:** 2022-08-23

**Authors:** Yu-Chin Liu, Yang-Hsiang Lin, Hsiang-Cheng Chi, Po-Shuan Huang, Chia-Jung Liao, Yu-Syuan Liou, Chiao-Chun Lin, Chia-Jung Yu, Chau-Ting Yeh, Ya-Hui Huang, Kwang-Huei Lin

**Affiliations:** 1 Department of Biochemistry, College of Medicine, Chang-Gung University, Taoyuan, Taiwan; 2 Graduate Institute of Biomedical Sciences, College of Medicine, Chang-Gung University, 259 Wen-Hwa 1 Road, Taoyuan, Taiwan, Republic of China; 3 Molecular Medicine Research Center, Chang Gung University, Taoyuan, Taiwan; 4 Liver Research Center, Chang Gung Memorial Hospital, Linkou, Taoyuan, Taiwan; 5 Graduate Institute of Integrated Medicine, China Medical University, Taichung, Taiwan; 6 Department of Cell and Molecular Biology, College of Medicine, Chang Gung University, Taoyuan, Taiwan; 7 Department of Thoracic Medicine, Chang Gung Memorial Hospital, Linkou, Taoyuan, Taiwan; 8 Research Center for Chinese Herbal Medicine, College of Human Ecology, Chang Gung University of Science and Technology, Taoyuan, Taiwan

**Keywords:** *CRNDE*, EGFR, Hepatocellular carcinoma (HCC), H3K9Ac, H3K27Ac, p300, C646, Sorafenib

## Abstract

**Background:**

Hepatocellular carcinoma (HCC) is one of the most common primary liver malignancies worldwide. The long-term prognosis for HCC remains extremely poor, with drug resistance being the major underlying cause of recurrence and mortality. The lncRNA colorectal neoplasia differentially expressed (*CRNDE*) is an epigenetic mediator and plays an important role to drive proliferation and drug resistance in HCC. However, *CRNDE* as an epigenetic regulator with influences sorafenib resistance in HCC is unclear. Thus, we explore the potential of targeting the *CRNDE*/p300/YY1 axis as a novel therapeutic strategy to overcome sorafenib resistance of HCC.

**Method:**

Detection of the expression level of *CRNDE* and *EGFR* in clinical specimens of HCC. *CRNDE*, EGFR, p300, and YY1expression were altered in HCC cells through transfection with different plasmids, and cell proliferation, migration, invasion, and sorafenib resistance were subsequently observed. Immunoprecipitation, chromatin immunoprecipitation, re-chromatin immunoprecipitation, site-directed mutagenesis, RNA Immunoprecipitation, immune fluorescence, qRT-PCR, and western blotting were performed to uncover the mechanisms of *CRNDE* regulation. The xenograft nude mice model was used to investigate the tumor growth and sorafenib resistance.

**Results:**

In this study, we showed that *CRNDE* expression is significantly positively correlated with that of epidermal growth factor receptor (*EGFR*) in clinical specimens of HCC and induces proliferation and sorafenib resistance of HCC via EGFR-mediated signaling. Mechanistically, *CRNDE* stabilized the p300/YY1 complex at the *EGFR* promoter and simultaneously enhanced histone H3K9 and H3K27 acetylation, which serve as markers of relaxed chromatin. EGFR was positively upregulated by the epigenetic complex, p300/YY1, in a manner dependent on *CRNDE* expression, leading to enhanced tumor cell proliferation and sorafenib resistance. Furthermore, C646, a p300 inhibitor, suppressed *EGFR* transcriptional activity by decreasing chromatin relaxation and YY1 binding, which effectively reduced proliferation/sorafenib resistance and prolonged overall survival.

**Conclusion:**

Our collective findings support the potential of targeting the *CRNDE*/p300/YY1 axis as a novel therapeutic strategy to overcome sorafenib resistance of HCC.

**Supplementary Information:**

The online version contains supplementary material available at 10.1186/s13148-022-01326-3.

## Introduction

Hepatocellular carcinoma (HCC), the predominant primary liver cancer and sixth most common cancer type worldwide [1], is the third leading cause of cancer-related death. High mortality, poor prognosis, and high-level recurrence of HCC are significantly associated with ineffective therapeutic management [2]. Sorafenib, a multi-kinase inhibitor, serves as effective first-line therapy that has successfully been used to extend the survival of advanced-stage HCC patients. However, the clinical benefits of sorafenib remain modest and most often consist of temporary tumor stabilization, thereby extending the overall median survival of patients [3]. To facilitate the development of effective therapeutic strategies, the mechanisms underlying sorafenib resistance require extensive clarification.

Dysregulation of lncRNAs in HCC can lead to sorafenib resistance [4]. Research to date has predominantly focused on the functions of lncRNAs as miRNA sponges regulating HCC target gene expression and mediating sorafenib resistance [5–7]. LncRNAs are involved in a number of other mechanisms, such as forming complexes regulating gene transcription by recruiting chromatin-modifying proteins to influence the epigenetic state, coordinating interactions between distal regulatory elements [8]. At present, the pathways by which lncRNAs influence sorafenib resistance are unclear. The lncRNA *CRNDE* was initially identified in colorectal cancer. *CRNDE* has been shown to act as an oncogene promoting tumor proliferation, invasion, migration, and drug resistance in multiple cancer types, including HCC [9–12]. A recent study showed that *CRNDE* induces sorafenib resistance in HCC by triggering autophagy via upregulation of ATG4B [13]. Additionally, *CRNDE* is reported to inhibit CELF2 and LATS2 expression and promote HCC proliferation, migration, and chemoresistance through epigenetic interactions with EZH2, a key component of the PRC2 complex. While accumulating evidence clearly indicates a role of *CRNDE* in drug resistance, the epigenetic molecular mechanisms by which *CRNDE* promotes resistance of HCC to sorafenib remain to be established. Accordingly, we focused on the mechanisms of action of *CRNDE* in sorafenib resistance of HCC and further evaluated the potential efficacy of epigenetic inhibitors in combating resistance.

The epidermal growth factor receptor EGFR is frequently overexpressed in the majority of HCC clinical samples and correlated with carcinogenesis [14] and therapeutic resistance to drugs (including sorafenib) [14, 15]. Notably, a monoclonal antibody directed against EGFR or RNA interference knockdown of EGFR has been shown to enhance the sensitivity of sorafenib-resistant HCC cell lines [3]. Clarification of the mechanisms affecting EGFR expression may therefore aid in effective management of sorafenib resistance in HCC.

Data from the current study confirmed dysregulation of *CRNDE*, with overexpression in tumors of HCC patients. *CRNDE* promoted HCC proliferation and sorafenib resistance through associations with the epigenetic regulator protein, p300, and transcription factor, YY1, and further regulation of chromatin relaxation to mediate *EGFR* expression and activity. Notably, treatment of HCC cells with a p300 inhibitor induced a significant decrease in proliferation and sorafenib resistance of HCC cells overexpressing *CRNDE*, supporting the involvement of epigenetic chromatin relaxation in *CRNDE*-mediated tumor growth and sorafenib resistance. In summary, we have uncovered a novel mechanism by which *CRNDE* mediates tumor growth and sorafenib resistance via interactions with p300 that may offer effective treatment avenues to combat sorafenib resistance in HCC.

## Materials and methods

### Human HCC specimens

Paired human HCC specimens were obtained from the Taiwan Liver Cancer Network (TLCN). Total RNA was reverse transcribed into cDNA and mRNA expression analyzed using qRT-PCR. The protocol was approved by the Medical Ethics and Human Clinical Trial Committee at Chang-Gung Memorial Hospital (IRB: 202000434B0).

### Cell lines and chemical reagents

Human hepatoma cell lines (HepG2, Mahlavu, Huh7, J7, Hep3B, and SkHep1) were routinely grown in DMEM supplemented with 10% fetal bovine serum. Cells were cultured at 37 °C in a humidified atmosphere of 95% air and 5% CO_2_. Sorafenib was purchased from Santa Cruz Biotechnology (Santa Cruz, CA, USA) and C636 and histone acetyltransferase inhibitor (HATi II) from Sigma-Aldrich (St Louis, MO, USA). All reagents were stored and used according to the manufacturer's instructions.

### Gene knockdown using short-hairpin RNA and siRNA

Lentivirus-based pLKO.1 plasmids expressing short-hairpin RNA (shRNA) targeting *CRNDE* were constructed following the guidelines of the RNAi Consortium (TRC) shRNA Design Process from the National RNAi Core Facility (Institute of Molecular Biology, Academia Sinica, Taipei, Taiwan). The shRNA sequence obtained was cloned into pLKO-TRC001. The oligonucleotides used for shRNA construction, shRNA clone list, and siRNA sequences are presented in Additional file 10: Table S1. Stable clones expressing shRNA plasmids via lentivirus were established in HCC cells.

### Generation of overexpression plasmids

The pcDNA3‐*CRNDE* expression plasmid was generated from the pcDNA3 vector using *Eco*RI and *Xho*I restriction sites. pLAS5w.PeGFP-I2-Bsd and pcDNA3.1/Myc-His A vectors were used to generate YY1-expressing pLAS5w.PeGFP-I2-Bsd-YY1 and pcDNA3.1/Myc-His A-YY1, respectively, followed by 48 h of incubation with DNA and TurboFect Reagent kit (Thermo Fisher Scientific, Waltham, MA, USA). All plasmids were prepared using an EasyPrep EndoFree Maxi Plasmid Extraction kit (DPT-BA17; Biotools Co., Ltd., Taipei, Taiwan).

### Quantitative RT-PCR

Quantitative reverse transcription PCR (qRT-PCR) was performed on total cellular RNA extracted with TRIzol reagent (Invitrogen, Waltham, MA, USA) according to the supplier's protocol (MAN0016385). The GenBank sequences and primer sequences are listed in Additional file 11: Table S2. Primers were designed with Primer3 Input (Life Technologies, Carlsbad, CA, USA).

### Chromatin immunoprecipitation (ChIP) and re-ChIP assay

Formaldehyde cross-linking and ChIP and re-ChIP assays were performed using a commercial kit [16] (Upstate Biotechnology, Lake Placid, NY). Chromatin was sonicated on ice to obtain DNA fragments of 0.3–1.5 kb (averaging 500 bp). A proportion of supernatant (20%) was used as total input control. To isolate a specific complex of interest, suitable antibodies were used to immunoprecipitate the chromatin components. Following removal of bound proteins, immunoprecipitated DNA was subjected to quantitative or regular PCR (35 cycles). Products amplified via regular PCR were separated on a 1.5% agarose gel and visualized via ethidium bromide staining. YY1 binding sites on the endogenous *EGFR* promoter were predicted using UCSC (https://genome.ucsc.edu/index.html; USA) and PROMO research (http://alggen.lsi.upc.es/cgi-bin/promo_v3/promo/promoinit.cgi?dirDB=TF_8.3). The primer sequences are listed in Additional file 12: Table S3. ChIP-quantitative PCR (qPCR) was performed using a specific kit and the same primer pairs. Relative quantification of IP products was performed using the △Ct method with normalization to control IgG. △Ct for each IP gene was calculated as ^2−△[Ct(IP)−Ct(input)] − 2−△[Ct(control IgG)−Ct(input)]^, with normalization of the relative level of DNA (in relation to input) specifically immunoprecipitated with YY1 (Santa Cruz Biotechnology, Santa Cruz, CA, USA) (sc-7341), p300 (61401), and H3K9Ac (61663) (Active Motif, Carlsbad, CA, USA) antibodies to that immunoprecipitated with control IgG. The primer sequences are listed in Additional file 12: Table S3.

### Cell death and proliferation analysis

Cells (3000 cells/well) in DMEM containing 10% FBS were used to seed 96-well plates for 24 h and incubated with sorafenib and C646 for a 3-day period. Cell viability was determined using the 3-(4,5-dimethylthiazol- 2-yl)-2,5-diphenyltetrazolium bromide (MTT) colorimetric assay. For the proliferation assay, growth rates were determined by staining with 0.4% Trypan blue solution using the LUNA Automated Cell Counter (Logos Biosystems, South Korea) at the indicated time points.

### Generation of luciferase-linked wild-type and mutant *EGFR* promoter constructs

The Renilla luciferase plasmid pGL3TK (2 μg; originally from Promega) kindly provided by our colleague, Professor J. Horng was co-transfected with each reporter plasmid for normalization. Transcriptional activity was monitored using a dual luciferase reporter assay (Promega) and a luminescence reader (LMaxII384, Molecular Devices, Sunnyvale, CA) according to the manufacturer's instructions. For each experiment, luciferase activity was normalized to that of Renilla luciferase. To generate transcription factor-binding mutants, putative binding sites were altered via site-directed mutagenesis (SDM) [17]. The primers used for the construction of mutant fragments are listed in Additional file 13: Table S4.

### Western blot analysis

Protein expression was determined as outlined previously [18]. Following electrophoresis and transfer, membranes were incubated with the appropriate primary antibodies (Additional file 14: Table S5) followed by goat anti-mouse or goat anti-rabbit horseradish peroxidase secondary antibodies (Amersham, Buckinghamshire, UK). Signals were visualized using enhanced chemiluminescence (ECL, Amersham).

### Co-immunoprecipitation (co-IP) assay

*CRNDE*-silenced and *CRNDE* overexpressing HCC cells were grown in 10 mm culture dishes (5 × 10^6^ cells/dish). Cells were transfected with pcDNA3.1/Myc-His A-YY1 expression plasmid prior to harvesting in lysis buffer. IP experiments were performed with antibodies against Myc (Abcam, Cambridge, MA, USA). Immunoprecipitated complexes were separated via 10% SDS-PAGE and subjected to western blot with the indicated antibodies.

### RNA immunoprecipitation (RIP)

The RIP assay was performed as described previously [19] using an antibody against p300 protein (Active Motif, Carlsbad, CA, USA).

### Immunohistochemistry

IHC staining was performed with specific primary antibodies (Additional file 14: Table S5) overnight at 4 °C as described previously [20]. Ki67 and *EGFR* were detected the next day using mouse antibody IgG conjugated to horseradish peroxidase (HRP; Santa Cruz Biotechnology) and diaminobenzidine (DAB). All sections were examined under an Olympus optical microscope equipped with a digital camera (Pixera, Santa Clara, CA, USA).

### Fractionation of nuclear and cytoplasmic RNA

Cells were washed twice with ice-cold PBS, followed by centrifugation for 5 min at 290 g and 4 °C. Cell pellets were resuspended in 1 mL RSB buffer (10 mM Tris–Cl (pH 7.4), 10 mM NaCl, and 3 mM MgCl_2_) for 3 min on ice. After further centrifugation at 1500 g for 3 min at 4 °C, the supernatant was discarded. Subsequently, cells were lysed via gentle resuspension in 150 μL RSBG40 (10 mM Tris–Cl (pH 7.4), 10 mM NaCl, 3 mM MgCl_2_, 10% glycerol, and 0.5% Nonidet P-40) and samples centrifuged at 4500 g for 3 min at 4 °C. The supernatant (cytoplasmic) fraction was collected in a new Eppendorf tube and 1 mL TRIzol reagent added to each sample (both the nuclear pellet and cytoplasmic supernatant) for extraction of RNA.

### Immunofluorescence staining

IF staining was performed according to a previous report [20]. Incubations were performed with primary rabbit anti-*EGFR* antibody (Cell Signaling Technology, Beverly, MA, USA) (1 μg/mL) overnight at 4 °C and secondary antibodies conjugated with Alexa-488 (1:200; Invitrogen) in PBS for 1 h at room temperature. Confocal laser scanning immunofluorescence microscopy (CLSM) was conducted using a Zeiss LSM510 META microscope (Carl Zeiss, Oberkochen, Germany). Images were analyzed using SM510 META Software and Adobe Photoshop 6.0.

### Animal models

In model I, nude mice were subcutaneously injected with pcDNA3-control or *CRNDE* overexpressing J7 (5 × 10^5^) cells. Tumor sizes were measured as described in a previous study [21]. In model II, nude mice were subcutaneously injected with pcDNA3-control or *CRNDE* overexpressing Huh7 (1 × 10^7^) cells and treated with different drugs (DMSO, C646, sorafenib and sorafenib plus C646). At a tumor volume of 60 mm^3^, tumors were intraperitoneally injected (IP) three times a week with DMSO or C646 (1 mg/kg) followed by oral administration of DMSO or sorafenib (30 mg/kg) twice a week for 30 days. Representative tumors were excised 43 days after the treatment regimen and sizes measured as described previously *t* [21]. Animal experiments were conducted according to the guidelines of United States National Institutes of Health and the Chang Gang Institutional Animal Care and Use Committee Guide for the Care and Use of Laboratory animals (Approval No.CGU109-021).

### Statistical analysis

Results were expressed as means ± s.d. of three independent experiments. Fisher’s exact or Mann–Whitney U test was used for between-group comparisons, where appropriate, and correlations between the results obtained with the two different methods analyzed with the Spearman’s test. Kaplan–Meier curves were constructed to analyze survival outcomes. Overall survival (OS) with death as an event was analyzed using the log-rank test. Statistical significance (*p* value) was calculated with the two-tailed Student's *t*-test for a single comparison between two groups. *p* values < 0.05 were considered significant.

## Results

### *CRNDE* is highly expressed in human HCC and positively correlated with tumor size, pathological stage, and poor survival

**Fig. 1:**
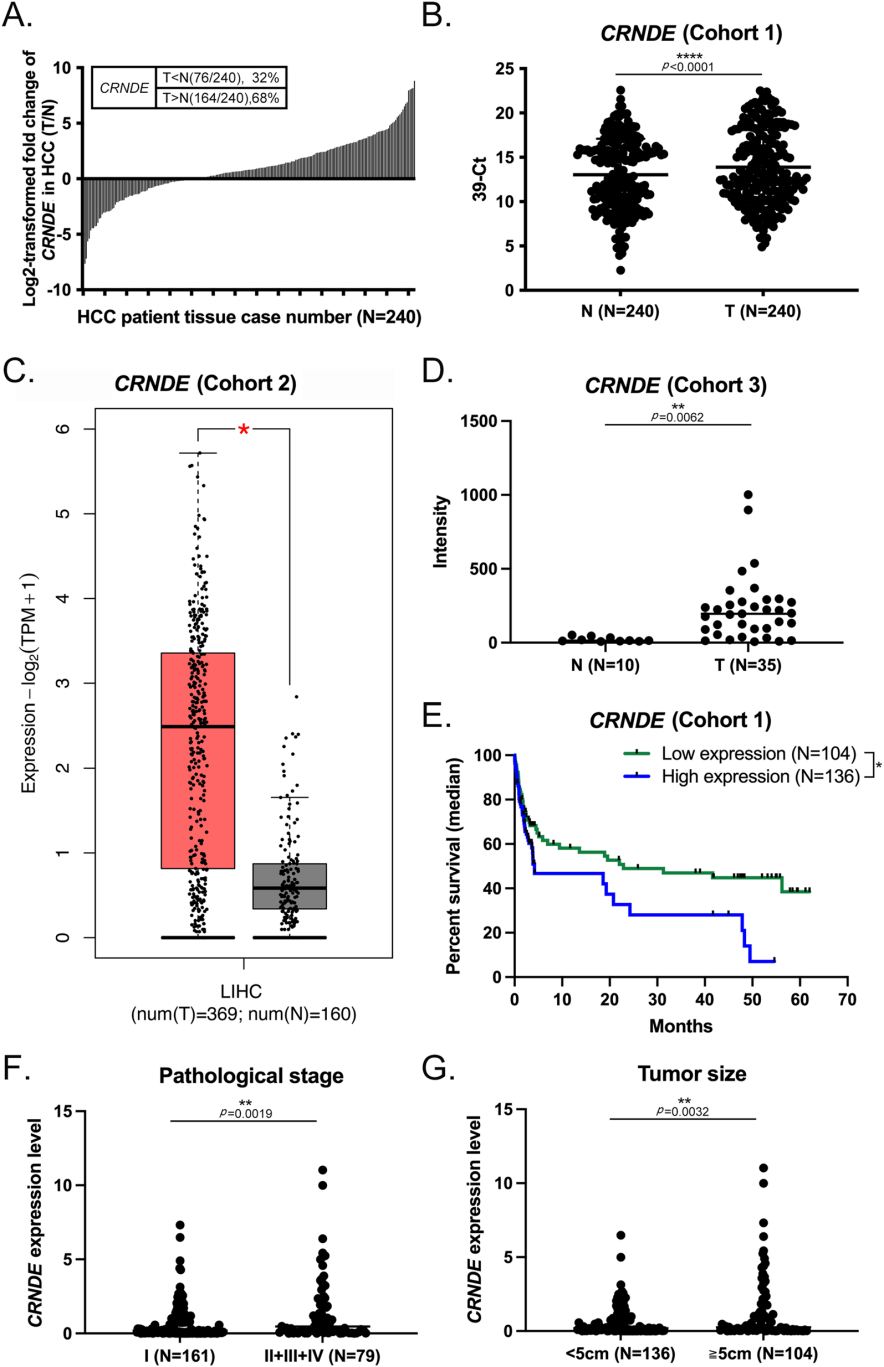
*CRNDE* is highly expressed in human HCC and positively correlated with tumor size, pathological stage, and poor survival. **A**, **B** High *CRNDE* expression in 240 pairs of human HCC tissues (*T*) relative to adjacent normal (*N*) tissues, determined using qRT-PCR with 18S rRNA as the loading control. Data are presented as fold change of *CRNDE* in human HCC and adjacent normal tissues. **C** Elevated levels of *CRNDE* RNA in human HCC (*T*) (*n* = 369) compared to adjacent normal tissue (*N*) (*n* = 160), calculated from The Cancer Genome Atlas datasets. **D** High expression of *CRNDE* in HCC (*n* = 35) compared with liver (*n* = 10) specimens from the Wurmbach Liver dataset in the Oncomine database. **E** Kaplan–Meier analysis of both median overall survival according to *CRNDE* expression. High *CRNDE* expression in human HCC tissues was associated with poor prognostic outcomes. (High *CRNDE* expression group: *CRNDE* expression values higher than the median; low *CRNDE* expression group: *CRNDE* expression values below the median; median = 2.034: tumor/normal (*T*/*N*) ratio median of *CRNDE* in 240 HCC specimens). **F**–**G** Data are presented as relative expression levels in tumor tissues. *CRNDE* expression was significantly increased in HCC patients with higher pathological stages and larger tumors. Statistical significance (*p* value) was calculated with the two-tailed Student's *t*-test for a single comparison between two groups. Data are presented as mean ± SD (*p < 0.05; **p < 0.01; ***p < 0.001; ****p < 0.0001)

*CRNDE* was upregulated in 240 paired in-house HCC specimens via qRT-PCR, compared with adjacent non-cancerous tissue, *CRNDE* was significantly upregulated (68%) in HCC tumor tissues (cohort 1, Fig. 1A). Similarly, *CRNDE* expression in HCC tumor tissue specimens was markedly higher relative to normal counterparts (cohort 1, Fig. 1B). These results were consistent with gene expression data from other publicly available datasets comprising several human hepatoma samples (cohort 2, The Cancer Genome Atlas data set; cohort 3, The Oncomine database from Wurmbach Liver Statistics), both showing high expression of *CRNDE* in HCC tumor tissues (Fig. 1C, D). Overall survival analysis of HCC patients further supported an association of high expression of *CRNDE* with shorter survival compared with low *CRNDE* expression (Fig. 1E). To further assess the significance of *CRNDE* expression in HCC, clinicopathological features were examined. Notably, *CRNDE* was increased at advanced pathological stage (cohort 1, Fig. 1F; *p* = 0.0019), larger tumors (cohort 1, Fig. 1G; *p* = 0.0032), virus infection (cohort 1, Additional file 1: Fig. S1A, HCV: *p* = 0.0124, HBV + HCV: *p* = 0.0008), and cirrhosis (cohort 1, Additional file 1: Fig. S1B, *p* = 0.0470). Our collective results support an oncogenic role of *CRNDE* in the biological progression of HCC.

### *CRNDE* promotes HCC tumorigenesis and sorafenib resistance

**Fig. 2:**
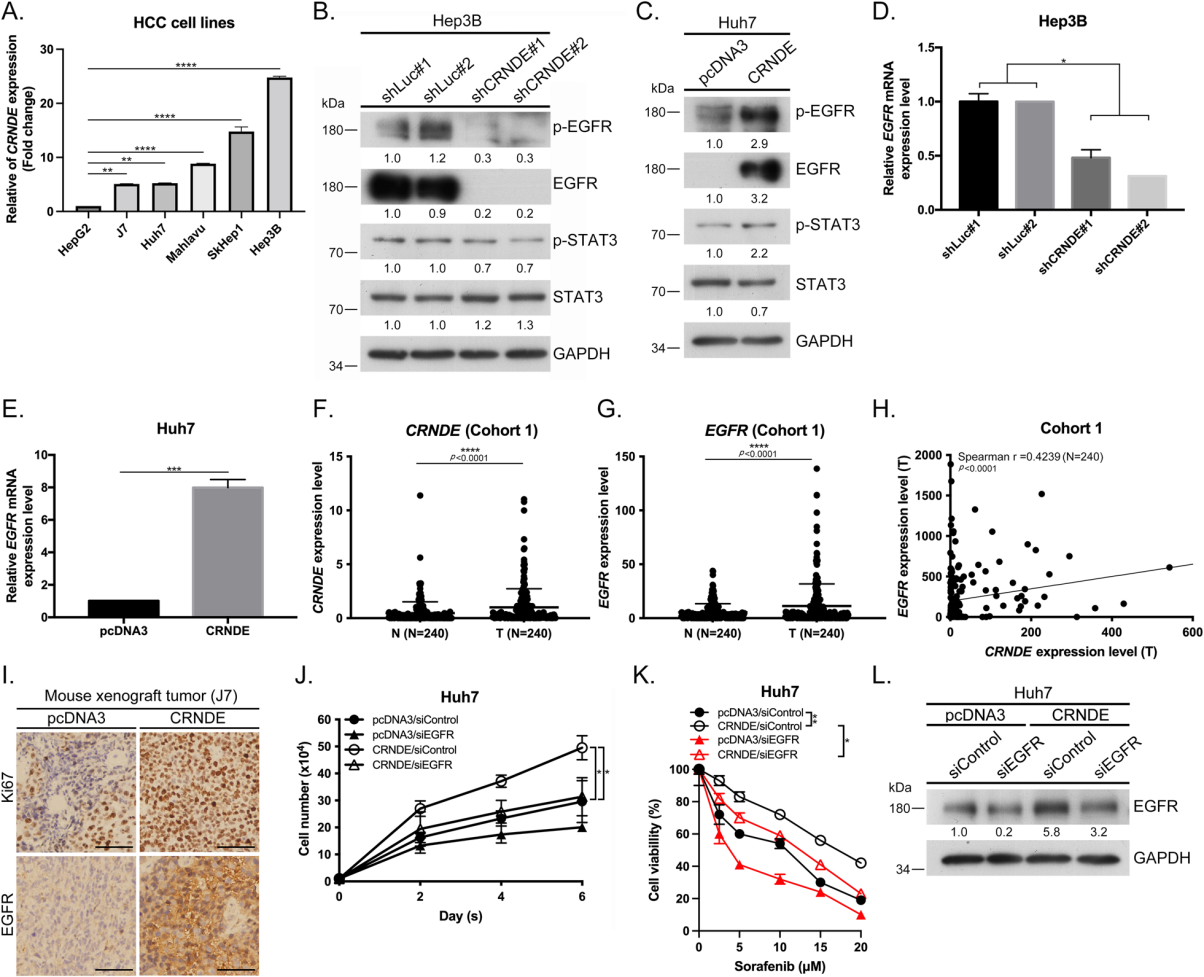
*CRNDE* is positively correlated with *EGFR* expression, thereby increasing proliferation and sorafenib resistance of HCC. **A** qRT-PCR analysis of *CRNDE* in various malignant compared with benign HCC cells (HepG2). **B**, **C** Western blot analysis of p-EGFR, EGFR, p-STAT3, and STAT3 protein levels in Hep3B cells (control and *CRNDE* depletion groups) and Huh7 cells (pcDNA3 and *CRNDE* overexpression groups). **D**, **E** qRT-PCR analysis of EGFR mRNA in Hep3B (control and *CRNDE* depletion groups) and Huh7 cells (pcDNA3 and *CRNDE* overexpression groups). **F** qRT-PCR analysis of *CRNDE* expression in 240 pairs of human HCC tissues (*T*) and adjacent normal (*N*) tissues. **G** qRT-PCR analysis of *EGFR* expression in 240 pairs of human HCC tissues (*T*) and adjacent normal (*N*) tissues. **H** Spearman correlation analysis of the clinical correlations between *CRNDE* and *EGFR* in HCC. **I** Representative IHC images of tumor xenografts stained with anti-Ki67 and anti-EGFR antibodies. Scale bar = 100 μm. **J** Huh7 cells were transfected with pcDNA3-control or *CRNDE* plasmids with siRNA-induced silencing of EGFR. Cell numbers were assessed after 48 h via the proliferation assay relative to control cells. **K** Huh7 cells transfected with pcDNA3-control and *CRNDE* overexpression plasmids with siRNA-induced silencing of EGFR were incubated with or without sorafenib and viability relative to control cells was assessed by MTT assay. **L** Western blot analysis of EGFR protein levels in Huh7 cells from pcDNA3-control and *CRNDE* overexpression groups with siRNA-induced silencing of EGFR. Statistical significance (*p* value) was calculated with the two-tailed Student's *t*-test for a single comparison between two groups. Data are presented as mean ± SD (*p < 0.05; **p < 0.01; ***p < 0.001)

We subsequently examined *CRNDE* expression in HCC cell lines. To this end, Hep3B and SkHep1 cells highly expressing *CRNDE* were selected for shRNA-based knockdown analysis (Fig. 2A) and *CRNDE* expression was induced in Huh7 and J7 cells expressing low levels of *CRNDE* via plasmid transfection. Knockdown of *CRNDE* significantly suppressed proliferation of both Hep3B and SkHep1 cells (Additional file 2: Fig. S2A, B). Conversely, *CRNDE* overexpression promoted Huh7 and J7 cell proliferation relative to cells expressing pcDNA3-control (Additional file 2: Fig. S2C, D). Simultaneously, in vivo experiments revealed that the final tumor volume was significantly greater in the *CRNDE* overexpression than the pcDNA3-control cell group (Additional file 2: Fig. S2E, F). Moreover, survival benefits were reduced under conditions of *CRNDE* overexpression, supporting an oncogenic role in HCC (Additional file 2: Fig. S2G). Additionally, *CRNDE* clearly suppressed migration and invasion of Hep3B and SkHep1 (Additional file 2: Fig. S2H, I, L, M). Conversely, migration and invasion abilities were enhanced in Huh7 and J7 cells overexpressing *CRNDE* (Additional file 2: Fig. S2J, K, N, O). In vivo suggested that the number of metastatic nodules in lungs was reduced in mice injected with *CRNDE*-silenced Hep3B (Additional file 2: Fig. S2P) and increased in livers of mice administered Huh7 overexpressing *CRNDE* (Additional file 2: Fig. S2Q). Silencing of *CRNDE* in Hep3B and SkHep1 cells promoted sensitization to sorafenib relative to the control shLuc group (Additional file 2: Fig. S2R, S) while *CRNDE* overexpression in Huh7 and J7 cells induced greater resistance to sorafenib relative to the corresponding pcDNA3-control groups (Additional file 2: Fig. S2T, U). These data are consistent with previous findings [11, 22, 23] that *CRNDE* is not only highly expressed but also markedly promotes tumorigenesis and sorafenib resistance in HCC specimens.

### *CRNDE* is positively correlated with upregulation of *EGFR*, thereby increasing proliferation and sorafenib resistance of HCC

Recent findings support an oncogenic role of *CRNDE* in glioma, whereby its upregulation is positively correlated with activation of EGFR signaling [24]. In our experiments, EGFR downstream signaling molecules (such as p-EGFR and p-STAT3) were reduced upon *CRNDE* silencing in Hep3B and SkHep1 cells (Figs. 2B, Additional file 3: Fig. S3A), and conversely, enhanced upon *CRNDE* overexpression in Huh7 and J7 cells (Figs. 2C, Additional file 3: Fig. S3B). Notably, suppression of *CRNDE* in HCC cells markedly reduced EGFR at both mRNA and protein levels while its overexpression exerted the opposite effect (Fig. 2B–E, Additional file 3: Fig. S3A–D). qRT-PCR analysis was further conducted to confirm the effects of *CRNDE* silencing and overexpression (Additional file 3: Fig. S3E–H). Compared with the pcDNA3-control group, higher green fluorescence intensity of EGFR was detected in the *CRNDE* overexpressing group via immunofluorescence staining (Additional file 3: Fig. S3I). Compared with adjacent non-cancerous tissue (N), *CRNDE* was significantly upregulated in HCC tumor tissues (T) (cohort 1, Fig. 2F). To validate upregulation of *EGFR* in HCC specimens, *EGFR* expression patterns were examined in 240 paired HCC specimens. Our qRT-PCR findings revealed significant expression of *EGFR* in HCC tumor tissues (T) (Fig. 2G). Additionally, the available online dataset suggested that the EGFR expression was no different between normal (N) and tumor (T) in cohort 2 and cohort 3 (Additional file 4: Fig. S3J and S3K). However, *EGFR* expression in HCC tumor tissue specimens was markedly higher relative to normal counterparts in our 240 paired HCC specimens (Fig. 2G), and *EGFR* was significantly increased at advanced pathological stage (cohort 1, Additional file 4: Fig. S3L; *p* = 0.0319), larger tumors (cohort 1, Additional file 4: Fig. S3M; *p* = 0.0346), and virus infection (cohort 1, Additional file 4: Fig. S3N; HBV: *p* = 0.0118, HCV: *p* < 0.0001, HBV + HCV: *p* < 0.0001). The Spearman correlation coefficient analysis confirmed a significant positive association of *CRNDE* with *EGFR* (Fig. 2H, Spearman *r*: 0.4239, *p* < 0.0001). Simultaneous in vivo experiments disclosed that both the number of Ki67-positive cells and EGFR expression were increased in *CRNDE* overexpression relative to pcDNA3-control-transfected J7 cells, as determined via IHC staining (Fig. 2I). We further assessed whether EGFR affects HCC cell proliferation and sorafenib resistance under *CRNDE* overexpression conditions. In both pcDNA3-control and *CRNDE* overexpressing Huh7 and J7 cell lines, knockdown of EGFR led to a significant reduction in cell proliferation (Fig. 2J, Additional file 4: Fig. S3O) and suppression of *CRNDE*-induced sorafenib resistance (Fig. 2K, Additional file 4: Fig. S3P). Western blot experiments were conducted to ascertain siRNA-mediated inhibition of EGFR protein in pcDNA3-control and *CRNDE* overexpressing Huh7 and J7 cells (Fig. 2L, Additional file 4: Fig. S3Q). Our results indicate that *CRNDE*-mediated enhancement of EGFR expression is involved in proliferation and sorafenib resistance of HCC cells.

### *CRNDE* upregulates *EGFR* through mediating chromatin relaxation in HCC

**Fig. 3:**
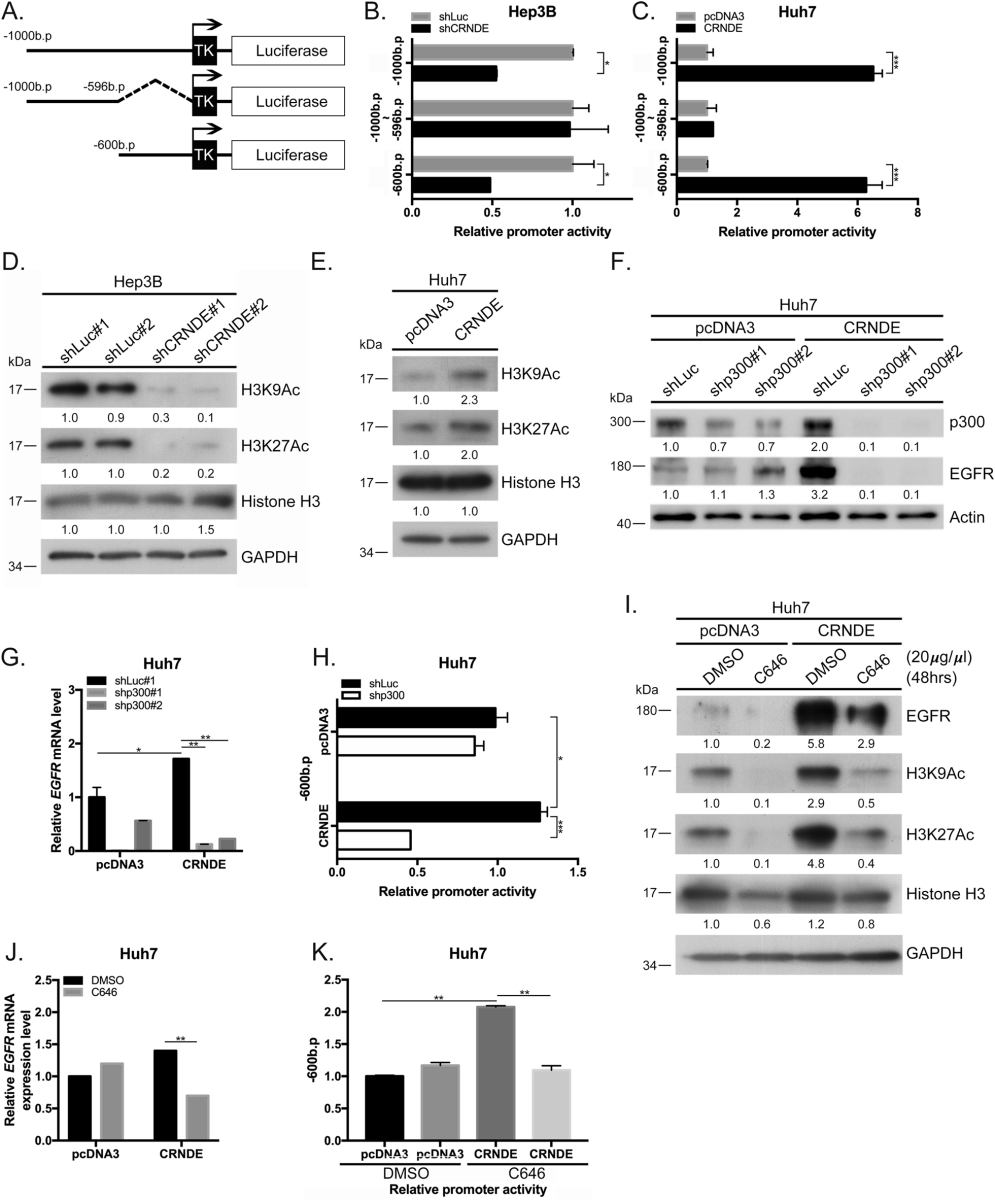
*CRNDE* upregulates EGFR through mediating chromatin relaxation in HCC. **A** Schematic representation of the *EGFR* promoter. Full-length *EGFR* promoter regions 600 bp and 1000 bp upstream of the transcriptional start site from the 5'-end of pGl3TK-EGFR. **B**, **C** Relative luciferase activities of each promoter were determined in Hep3B under conditions of *CRNDE* depletion and Huh7 under conditions of *CRNDE* overexpression using the reporter assay. **D**, **E** Western blot analysis of H3K9Ac, H3K27Ac, and histone H3 protein levels in Hep3B (control and *CRNDE* depletion groups) and Huh7 cells (pcDNA3 and *CRNDE* overexpression groups). **F**, **G** Western blot and qRT-PCR analysis of p300 and EGFR protein and *EGFR* mRNA in Huh7 cells under *CRNDE* overexpression and shRNA-induced silencing of p300, respectively. **H** Relative luciferase activity of the *EGFR* promoter in Huh7 cells with *CRNDE* overexpression or shRNA-silenced p300, determined using the reporter assay. **I** Huh7 cells transfected with pcDNA3-control or *CRNDE* overexpression plasmids were incubated with DMSO or C646 and EGFR, H3K9Ac, H3K27Ac, and histone H3 protein levels were assessed by western blot. **J** qRT-PCR analysis of *EGFR* mRNA levels. **K** Huh7 cells transfected with pcDNA3-control or *CRNDE* overexpression plasmids were incubated with DMSO or C646 and relative luciferase activities of the promoter were assessed by the reporter assay. Statistical significance (*p* value) was calculated with the two-tailed Student's *t*-test for a single comparison between two groups. Data are presented as mean ± SD (*p < 0.05; **p < 0.01; ***p < 0.001)

To establish the mechanisms by which *CRNDE* promotes *EGFR* mRNA transcription, the promoter assay was performed. The activity of the region 600 bp upstream of the promoter (Fig. 3A) was significantly reduced upon *CRNDE* silencing in Hep3B and SkHep1 cells (Fig. 3B, Additional file 5: Fig. S4A), and conversely, enhanced with *CRNDE* overexpression in Huh7 and J7 cells (Fig. 3C, Additional file 5: Fig. S4B). Subcellular fractionation analysis using *GAPDH* and *U1* as cytoplasmic and nuclear localization markers, respectively, showed that *CRNDE* primarily localizes in the nucleus of HCC cells (Additional file 5: Fig. S4C, D). Acetylated H3K27 and H3K9 are associated with alterations in transcription regulation [25, 26]. High H3K27Ac expression at the endogenous *EGFR* promoter was predicted using the UCSC Genome Browser (https://genome.ucsc.edu/). To further ascertain whether *CRNDE* is involved in the regulation of *EGFR* gene expression via chromatin relaxation, we examined the status of representative histone modifications. Acetylated H3K9 and H3K27 signals were reduced upon *CRNDE* silencing in Hep3B and SkHep1 cells (Fig. 3D, Additional file 5: Fig. S4E). Conversely, *CRNDE* overexpression enhanced acetylated H3K9 and H3K27 signals relative to control Huh7 and J7 cells (Fig. 3E, Additional file 5: Fig. S4F). Recent studies have shown that H3K9 and H3K27 are acetylated by p300, a member of the histone acetyltransferase family [27]. Accordingly, we investigated whether p300 is involved in the mediation of EGFR expression by *CRNDE*. Interestingly, knockdown of p300 inhibited EGFR protein and mRNA expression under *CRNDE* overexpression conditions in Huh7 cells (Fig. 3F, G) but had no effect in the corresponding pcDNA3-control group. Additionally, suppression of p300 reduced *EGFR* promoter activity in *CRNDE* overexpressing HCC cells (Fig. 3H, Additional file 5: Fig. S4G). To determine whether *EGFR* is transcriptionally activated by chromatin relaxation via p300 acetyltransferase activity mediated by *CRNDE*, the effect of C646 (a p300 acetyltransferase inhibitor [28, 29]) on EGFR expression was evaluated under *CRNDE* overexpression conditions. EGFR, H3K9Ac, and H3K27Ac protein signals were significantly induced in *CRNDE* overexpressing relative to the control cell groups. Notably, treatment with C646 led to a marked reduction in expression levels of these proteins (Fig. 3I, Additional file 5: Fig. S4H). Furthermore, C646 suppressed *EGFR* mRNA levels to a significant extent in Huh7 and J7 cells overexpressing *CRNDE* (Fig. 3J, Additional file 5: Fig S4I). In the luciferase reporter assay, treatment with C646 reduced *EGFR* promoter activity compared to that in DMSO under *CRNDE* overexpression (Fig. 3K, Additional file 5: Fig. S4J). These results suggest that *CRNDE* induces EGFR upregulation by increasing histone acetylation through the regulation of p300.

### YY1 promotes HCC tumorigenesis via induction of *EGFR* transcription activity under conditions of *CRNDE* overexpression

**Fig. 4:**
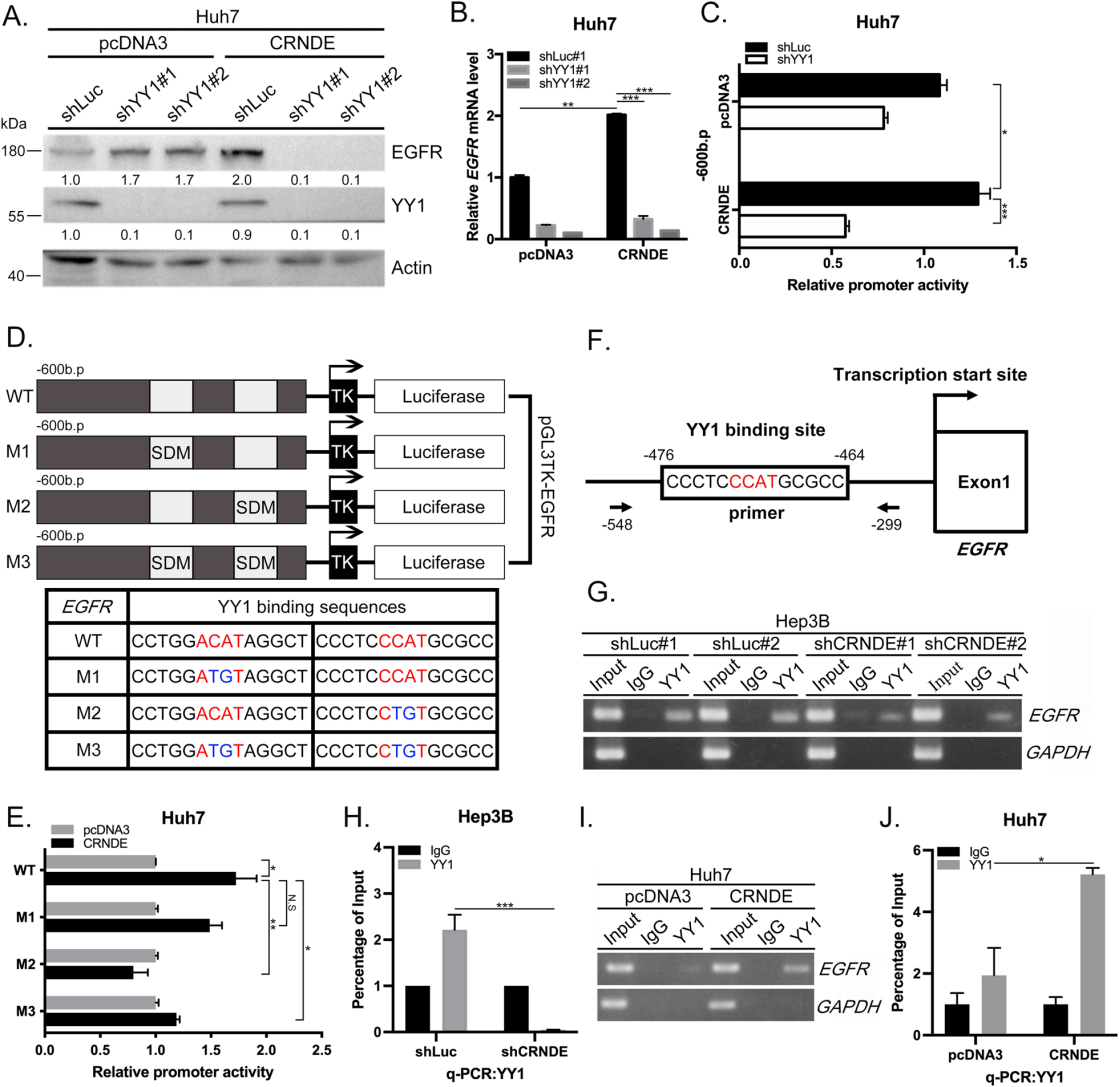
YY1 promotes HCC tumorigenesis via induction of EGFR transcription activity under conditions of *CRNDE* overexpression. **A**, **B** Western blot and qRT-PCR analysis of YY1 and EGFR protein and *EGFR* mRNA expression in Huh7 cells under conditions of *CRNDE* overexpression and silencing of YY1, respectively. **C** Relative luciferase activities of the *EGFR* promoter in Huh7 cells with *CRNDE* overexpression and shRNA-silenced YY1 were determined using the reporter assay. **D** Schematic representation of YY1 binding sites in the 0.6 kb *EGFR* promoter region. Partial wild-type (WT) and mutant YY1 binding sequences are listed below, including mutant YY1 at binding site 1 (M1), mutant YY1 at binding site 2 (M2), and double mutant YY1 at binding sites 1 and 2 (M3). **E** Reporter assay of the relative luciferase activity of each promoter in Huh7 cells transfected with pcDNA3-control or *CRNDE* overexpression constructs. Luciferase activities of the *EGFR* promoter with mutant YY1 binding site 1 (M1), YY1 binding site 2 (M2), and YY1 binding site 1 and 2 (M3), taking wild-type (WT) *EGFR* promoter activity as 100%. **F** Schematic diagram of the promoter and other regions of the *EGFR* gene. The YY1 binding site and transcriptional initiation site (+ 1) are indicated. YY1 binding to the promoter region of *EGFR*, ranging from 464 to 476 bp upstream of the transcription start site, and a 249-bp PCR product containing the YY1-binding site on the *EGFR* promoter are shown. The positions of PCR primers and elements relative to the transcription initiation site are numbered. **G**–**J** YY1 binding to the *EGFR* promoter in Hep3B (control and *CRNDE* depletion) and Huh7 (pcDNA3 and *CRNDE* overexpression) cells was determined via ChIP PCR or ChIP-q-PCR. Images of representative gels of ChIP PCR products. The chromatin of cells subjected to the indicated treatments was immunoprecipitated with YY1 antibody or IgG control. Co-IP DNA for the YY1 binding region (*EGFR*) or negative control region (*GAPDH*) was detected using PCR. Statistical significance (*p* value) was calculated with the two-tailed Student's t-test for a single comparison between two groups. Data are presented as mean ± SD (*p < 0.05; **p < 0.01; ***p < 0.001)

We identified a series of transcription factors with binding sequences in the 600-bp region upstream of the promoter via the PROMO database (http://alggen.lsi.upc.es/cgi-bin/promo_v3/promo/promoinit.cgi?dirDB=TF_8.3) prediction, including C/EBPβ [30], c-myc [31], STAT3 [32], and YY1 [33], which could also interact with p300. To establish whether these transcription factors are involved in the regulation of *EGFR* in response to *CRNDE*, specific shRNAs were used for their knockdown in Huh7 cells overexpressing *CRNDE*. Interestingly, only the knockdown of YY1 inhibited the *CRNDE*-induced increase in *EGFR* mRNA (data not shown). Consistently, *CRNDE*-induced EGFR protein was inhibited upon YY1 silencing (Fig. 4A, B; Additional file 6: Fig. S5A, B), along with *EGFR* transcription activity (Fig. 4C, Additional file 6: Fig. S5C). The PROMO database predicts that the 600-bp region upstream of the promoter potentially contains two YY1 binding sites. Next, we designed variants of the YY1 binding sites in the *EGFR* promoter via site-directed mutagenesis (SDM), designated M1, M2, and M3 (Fig. 4D). *EGFR* promoter activity was inhibited in M2 and M3 variants under conditions of *CRNDE* overexpression (Fig. 4E, Additional file 6: Fig. S5D), supporting the involvement of YY1 in the transcriptional activation of *EGFR* by *CRNDE*. The ChIP assay was conducted to confirm YY1 binding to the promoter region of *EGFR*, ranging from 464 to 476 bp upstream of the transcription start site. A 249-bp PCR product containing the YY1-binding site on the *EGFR* promoter was obtained. The locations and sequences of PCR primers are depicted in Fig. 4F. YY1 binding was reduced upon *CRNDE* silencing in Hep3B and SkHep1 cells (Fig. 4G, H; Additional file 6: Fig. S5G). *GADPH* was used as a negative control (Additional file 6: Fig. S5E). Conversely, *CRNDE* overexpression in Huh7 and J7 (Fig. 4I, J; Additional file 6: Fig. S5H; *GADPH* as a negative control, Additional file 6: Fig. S5F) cells enhanced YY1 binding to the *EGFR* promoter region. Based on these findings, we conclude that *EGFR* transcriptional activity is induced by enhancing specific YY1 binding to its promoter region following *CRNDE* expression.

### *CRNDE* regulates H3K9Ac/p300/YY1 complex binding on the *EGFR* promoter

**Fig. 5:**
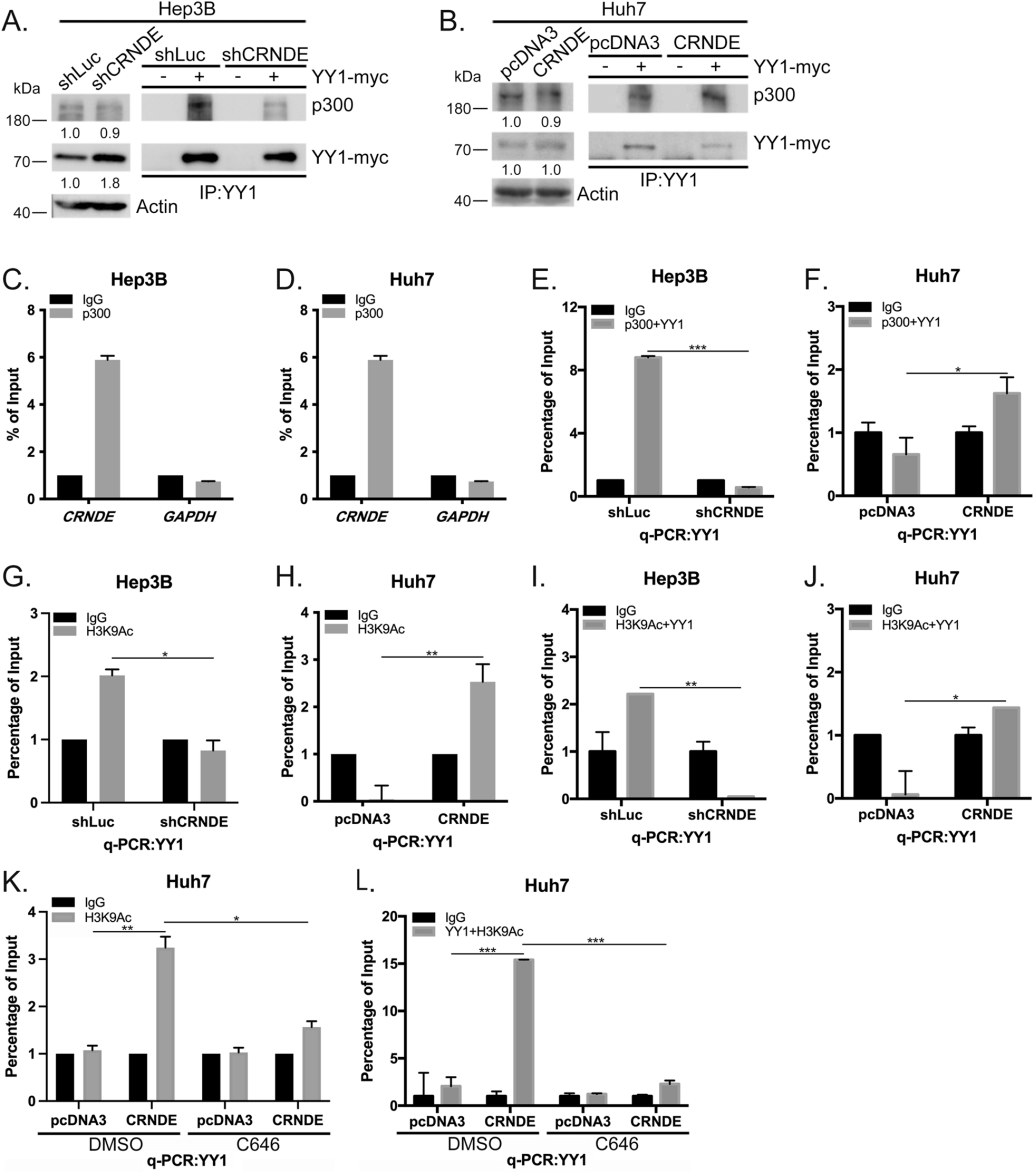
*CRNDE* regulates H3K9Ac/p300/YY1 complex binding on the EGFR promoter. **A**, **B** In Hep3B cells transfected with YY1-myc under control and *CRNDE* depletion conditions and Huh7 cells transfected with YY1-myc under control and *CRNDE* overexpression conditions, p300 and YY1-myc association were detected via the co-immunoprecipitation (co-IP) assay. **C**, **D** Hep3B and Huh7 extracts were immunoprecipitated using mouse IgG or p300 antibodies. Immunoprecipitation of p300-associated RNA was detected via qRT-PCR using *GAPDH* as a negative control. **E**, **F** p300/YY1 complex binding to the *EGFR* promoter in Hep3B (control and *CRNDE* depletion) and Huh7 (pcDNA3 and *CRNDE* overexpression) cell groups were determined with the re-ChIP assay as described in the Materials and Methods section of Supplementary Information. **G**, **H** H3K9Ac at the *EGFR* promoter in Hep3B (control and *CRNDE* depletion) and Huh7 (pcDNA3 and *CRNDE* overexpression) cell groups were determined with the ChIP assay. **I**, **J** H3K9Ac/YY1 complex binding to the *EGFR* promoter in Hep3B (control and *CRNDE* depletion) and Huh7 (pcDNA3 and *CRNDE* overexpression) cell groups was determined with the re-ChIP assay. **K** H3K9Ac binding to the EGFR promoter in Huh7 cells with *CRNDE* overexpression in the presence or absence of C646 determined with the ChIP assay. **L** H3K9Ac/YY1 binding to the *EGFR* promoter in Huh7 cells with *CRNDE* overexpression in the presence or absence of C646 determined with the re-ChIP assay. Statistical significance (*p* value) was calculated with the two-tailed Student's *t*-test for a single comparison between two groups. Data are presented as mean ± SD (*p < 0.05; **p < 0.01; ***p < 0.001)

p300 can act as both a coactivator and corepressor through interactions with YY1 and modulation of YY1-mediated transcriptional regulation [33]. Here, we further examined the hypothesis that p300 associates with YY1 as a transcriptional complex that regulates *EGFR* under conditions of *CRNDE* overexpression. Data from the co-immunoprecipitation (co-IP) assay revealed decreased association of p300 with YY1 following *CRNDE* silencing in Hep3B (Fig. 5A). Conversely, *CRNDE* overexpression in Huh7 promoted the binding of p300 and YY1 (Fig. 5B). The RNA immunoprecipitation (RIP) assay confirmed the association of p300 with *CRNDE* in HCC (Fig. 5C, D; Additional file 7: Figs. S6A, B). These results suggest that p300 is not only associated with *CRNDE* in HCC but also further stimulates associations with YY1 under conditions of *CRNDE* overexpression. Furthermore, we observed decreased binding of p300/YY1 to the *EGFR* promoter under *CRNDE* silencing in the re-ChIP assay (Fig. 5E, Additional file 7: Fig. S6C; negative control *GADPH*, Additional file 7: Fig. S6D, E). Conversely, *CRNDE* overexpression led to elevated binding to the *EGFR* promoter (Fig. 5F, Additional file 7: Fig. S6F; negative control *GADPH*, Additional file 7: Fig. S6G, H). To further determine whether *CRNDE* influences p300/YY1 binding to the *EGFR* promoter through chromatin relaxation, histone markers were assayed. Initially, H3K9Ac binding to the *EGFR* promoter was markedly reduced upon *CRNDE* silencing (Fig. 5G, Additional file 7: Fig. S6I; negative control *GADPH*, Additional file 7: Fig. S6J, K) while *CRNDE* overexpression led to increased binding to the *EGFR* promoter (Fig. 5H, Additional file 6: Fig. S5L; negative control *GADPH*, Additional file 7: Fig. S6M, N). Data from the further re-ChIP analysis revealed reduced H3K9Ac/YY1 binding to the *EGFR* promoter upon *CRNDE* silencing (Fig. 5I, Additional file 7: Fig. S6O; negative control *GADPH*, Additional file 7: Fig. S6P, Q). Conversely, overexpression of *CRNDE* dramatically stimulated these interactions (Fig. 5J, Additional file 7: Fig. S6R; negative control *GADPH*, Additional file 7: Fig. S6S, T). However, no changes in the binding ability of H3K27Ac/YY1 to the *EGFR* promoter under conditions of *CRNDE* expression were observed in the re-ChIP assay (data not shown). Finally, interactions of both H3K9Ac and YY1/H3K9Ac to the *EGFR* promoter were enhanced under *CRNDE* overexpression, as determined from ChIP and re-ChIP analyses, and reduced following C646 treatment (Fig. 5K, L; negative control *GADPH*, Additional file 7: Fig. S6U, V). The collective results suggest that *CRNDE* mediates both chromatin relaxation and H3K9Ac/p300/YY1 complex binding at the *EGFR* promoter region to regulate *EGFR* transcriptional activity.

### *CRNDE* promotes CDX tumor growth in mice via p300/YY1/H3K9Ac regulation of EGFR

**Fig. 6:**
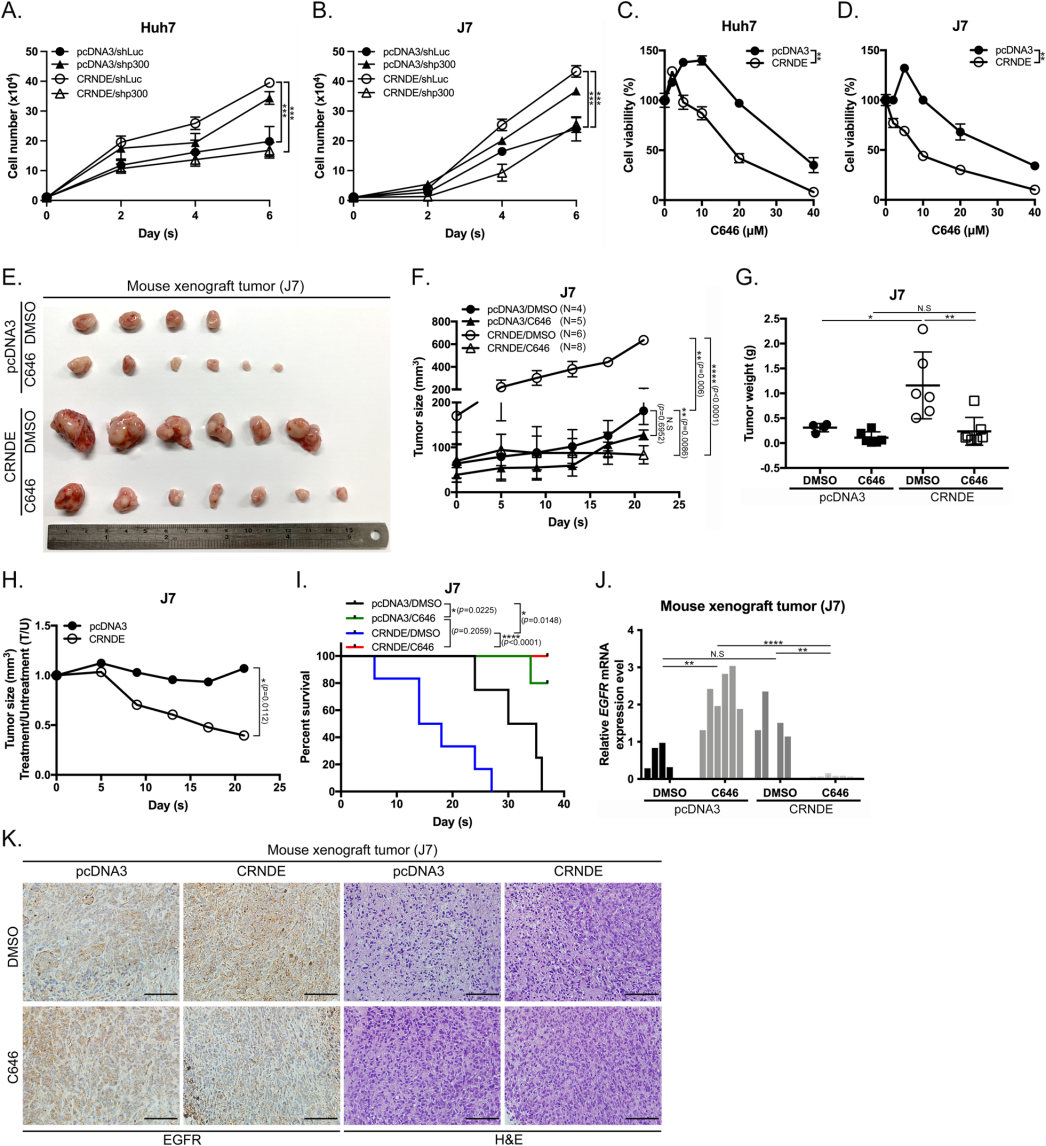
*CRNDE* promotes CDX tumor growth in mice via p300/YY1/H3K9Ac regulation of EGFR. **A**, **B** Huh7 and J7 cells were transfected with pcDNA3-control or pcDNA3-*CRNDE* plasmids with shRNA-silenced p300. Cell numbers were assessed with the proliferation assay. **C**, **D** Huh7 and J7 cells from pcDNA3-control and *CRNDE* overexpression groups were incubated with or without C646 and evaluated via the MTT assay. **E** Nude mice were subcutaneously injected with pcDNA3-control or *CRNDE* overexpressing J7 cells followed by vehicle control (DMSO) or C646 treatment and tumor growth monitored. Tumor sizes (cm) are indicated at the bottom. **F**, **G** Chart showing tumor sizes and weights from **E**. **H** Kinetic changes in tumor sizes of CDX following C646 treatment. **I** Survival rates of nude mice from **E**. Kaplan–Meier plots using standard tumor sizes (150 mm^3^) as a criterion for euthanasia. **J** qRT-PCR analysis of *EGFR* mRNA in tumors of mice. **K** Determination of EGFR protein expression in mouse tumors via immunohistochemistry. Positive staining (brown) in tumor tissues. Size reference (10 μm) is indicated. Statistical significance (*p* value) was calculated with the two-tailed Student's *t*-test for a single comparison between two groups. Data are presented as mean ± SD (*p < 0.05; **p < 0.01; ***p < 0.001; ****p < 0.0001)

In HCC specimens, p300 enhances proliferation via epigenetic regulation of gene transcription [34–36]. Here, we assessed whether the promontory effects of p300 on HCC cell proliferation are related to *CRNDE*. Proliferation was significantly reduced in *CRNDE* overexpressing Huh7 and J7 cells and conversely, increased in pcDNA3-control cells with knockdown of p300 (Fig. 6A, B). Additionally, both migration and invasion abilities were increased in Huh7 cells with *CRNDE* overexpression and inhibited followed by knockdown of p300 or YY1 (Additional file 8: Fig. S7A, B). Quantification of Huh7 cell numbers using the transwell assay is presented in the right panel (Additional file 8: Fig. S7C, D). The collective results suggest that *CRNDE* influences migration, invasion, and proliferation of HCC cells through p300 and YY1-mediated. Recent studies have shown that C646 effectively reduces the proliferation of HCC cells [35, 37, 38]. *CRNDE* overexpressing Huh7 and J7 cells exposed to C646 for 72 h displayed decreased viability in a dose-dependent manner compared to the pcNDA3-control groups (Fig. 6C, D). We further examined the potential therapeutic effect of C646 on HCC xenografts in nude mice. To this end, two groups of mice were subcutaneously injected with J7 cells (transfected with pcDNA3-control or *CRNDE* overexpressing constructs). *CRNDE* overexpressing J7 cells induced a significant increase in tumor sizes examined on day 5 following injection. A representative tumor sample is depicted in Fig. 6E. Final tumor sizes were considerably greater in the *CRNDE* overexpressing than pcDNA3-control cell-injected groups (Fig. 6F). C646 treatment for 21 days induced the strongest inhibition of tumor growth, resulting in markedly smaller tumor sizes and weights in *CRNDE* overexpressing cancer cell line-derived xenograft (CDX) mice (Fig. 6G, H). As expected, the survival curve for the *CRNDE* overexpression group treated with C646 at the sub-pharmacologic dose was longer than that for untreated mice (Fig. 6I). Additionally, *EGFR* mRNA levels were significantly increased upon *CRNDE* overexpression and conversely, decreased following treatment with C646 (Fig. 6J). Consistently, IHC analysis revealed marked inhibition of EGFR protein expression in cells treated with C646 for 21 days compared to untreated cells overexpressing *CRNDE* (Fig. 6K). The collective results support the utility of the p300 inhibitor in suppressing tumor growth under conditions of *CRNDE* expression.

### *CRNDE* enhances sorafenib resistance through upregulation of EGFR with p300 in HCC

**Fig. 7:**
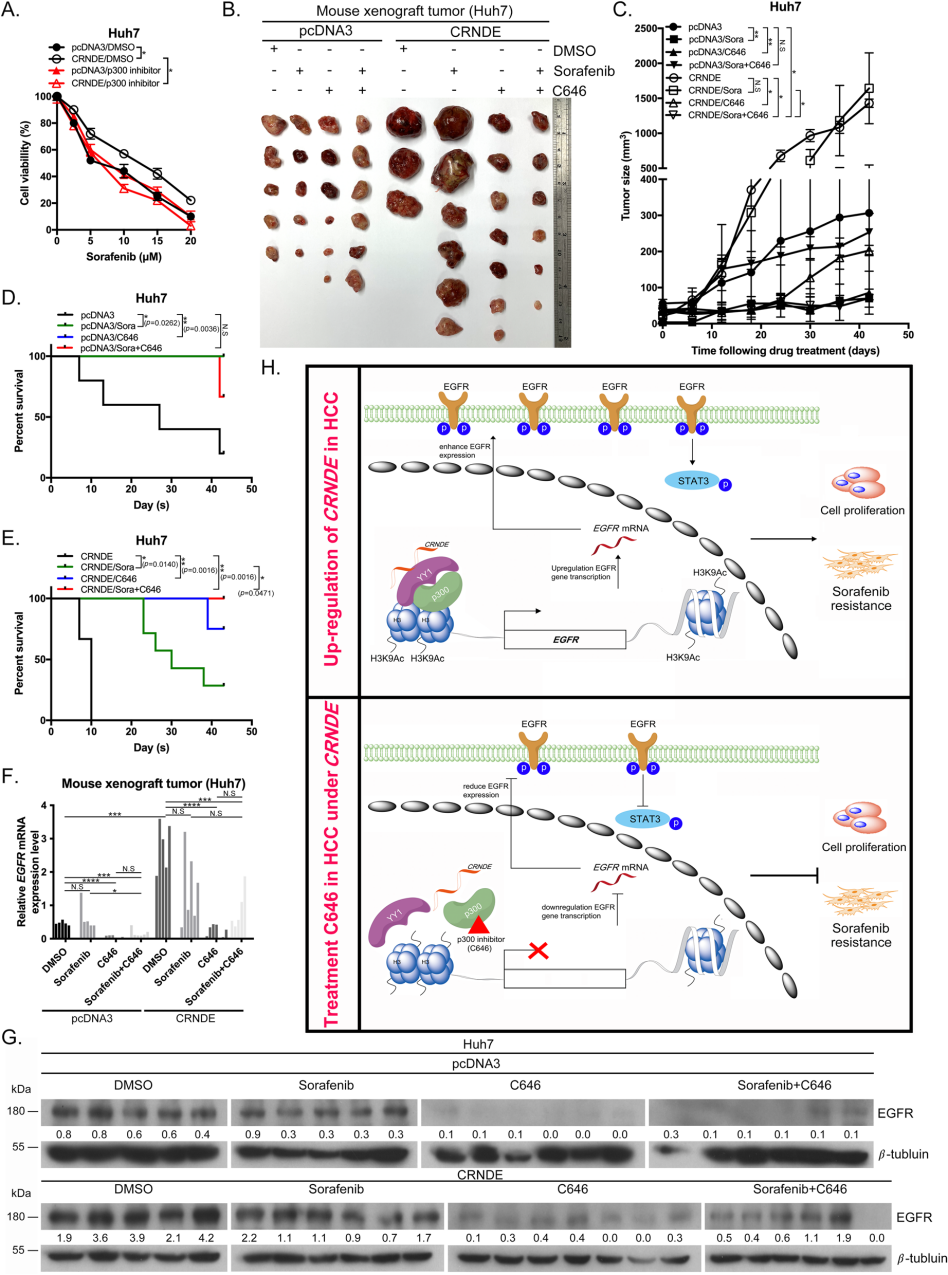
*CRNDE* enhances sorafenib resistance through upregulation of EGFR with p300 in HCC. **A** Huh7 cells from pcDNA3-control and *CRNDE* overexpression groups were incubated with or without sorafenib and assessed via the MTT assay. **B** Nude mice were subcutaneously injected with pcDNA3-control or *CRNDE* overexpression Huh7 cells, followed by DMSO (vehicle control), C646 or sorafenib treatment, and tumor growth monitored. Tumor sizes (cm) are indicated at the bottom. **C** Chart showing tumor sizes and weights from **B**. **D**, **E** Survival rates of nude mice from **B**. Kaplan–Meier plots using the standard tumor size (200 mm^3^) as a criterion for euthanasia from pcDNA3-control and *CRNDE* overexpression groups. **F**, **G** qRT-PCR and western blot analyses of *EGFR* mRNA and protein expression in tumors of mice. **H** Working model of the function of the *CRNDE*/p300/H3K9Ac/YY1 complex in regulation of EGFR expression. Statistical significance (*p* value) was calculated with the two-tailed Student's *t*-test for a single comparison between two groups. Data are presented as mean ± SD (*p < 0.05; **p < 0.01; ***p < 0.001; ****p < 0.0001)

The influence of p300 inhibitors on sorafenib resistance in HCC has not been extensively investigated to date. Based on the finding that C646 is effective against HCC in vitro and in vivo (Fig. 6), we further examined whether C646 could reduce cell viability in vitro upon co-treatment with sorafenib in *CRNDE* overexpressing Huh7 and J7 cells. Notably, sorafenib resistance was reduced in C646-treated *CRNDE* overexpressing Huh7 and J7 cells in a dose-dependent manner (Fig. 7A, Additional file 9: Fig. S8A). To assess the effects of C646 on sorafenib resistance of *CRNDE* overexpressing HCC cells in vivo, a mouse xenograft model was used. *CRNDE* overexpressing Huh7 xenografts displayed elevated tumor sizes compared to those expressing pcDNA3-control (Fig. 7B, C). Simultaneously, sorafenib resistance was enhanced under *CRNDE* overexpression, with no significant decrease in tumor size following sorafenib treatment relative to the pcNDA3-control (Fig. 7B, C). Only the pcNDA3-control group showed a marked reduction in tumor size following treatment with sorafenib (Fig. 7B, C). Under conditions of *CRNDE* overexpression, combined treatment with C646 and sorafenib for 21 days dramatically reduced resistance compared to treatment with sorafenib only (Fig. 7B–D). However, in the presence of C646 only, HCC tumor size was equivalent to that recorded following treatment with a combination of C646 and sorafenib under conditions of *CRNDE* overexpression (Fig. 7B, C). In terms of survival curves, pcDNA3-control mice died within 43 days, with a median survival of 27 days, while mice in the *CRNDE* overexpression group died within 10 days, with a median survival of 10 days (Fig. 7D, E). Treatment of pcDNA3-control mice with either sorafenib or C646 led to increased survival times (Fig. 7D). Sorafenib treatment under *CRNDE* overexpression conditions increased survival, with a median survival time of 30 days (Fig. 7E). Notably, C646-treated or sorafenib and C646-treated mice displayed significantly longer survival times compared to non-treated or sorafenib only groups (Fig. 7E). *EGFR* mRNA and protein expression were significantly induced upon *CRNDE* overexpression and reduced by both C646 alone and in combination with sorafenib (Fig. 7F, G). These results support a critical role of *CRNDE* in sorafenib resistance and suggest that the p300 inhibitor C646 exerts inhibitory and additive effects on sorafenib resistance to prolong overall survival through downregulation of EGFR expression.

## Discussion

Currently, sorafenib is the most effective clinical drug for treatment of advanced HCC, but progressive therapy with this drug is often associated with emergence of resistance. Therefore, only 30% of HCC patients effectively benefit from sorafenib treatment. The mechanisms underlying sorafenib resistance remain to be established. Here, we have demonstrated an oncogenic role of *CRNDE* in sorafenib resistance of HCC, consistent with previous reports [13]. In our experiments, *CRNDE* was upregulated in HCC tumor specimens and induced elevation of *EGFR* expression, thereby increasing proliferation and sorafenib resistance. Mechanistically, *CRNDE* enhanced p300/YY1 binding to the *EGFR* promoter and simultaneously increased H3K9Ac mark expression at the promoter region, resulting in marked transcriptional activation of *EGFR* via the mediation of chromatin relaxation. Treatment with the p300 inhibitor C646 induced repression of *EGFR* transcription activity under *CRNDE* overexpression, with consequent suppression of HCC proliferation and enhancement of sensitivity to sorafenib, leading to additive effects in prolonging overall survival (Fig. 7H). Based on these findings, we propose that EGFR is positively upregulated by the epigenetic p300/YY1 complex in a manner dependent on *CRNDE* expression, thereby leading to sorafenib resistance. The regulatory role of *CRNDE* in the therapeutic resistance of HCC uncovered in this study offers a novel therapeutic avenue to combat tumor resistance to sorafenib.

Earlier studies have shown that EGFR signaling promotes sorafenib resistance in HCC, which could be effectively reversed by EGFR signaling inhibitors or siRNA knockdown of EGFR [3]. Additionally, *CRNDE* promotes glioma cell growth through the enhancement of the activation status of the EGFR pathway [24]. We observed that *CRNDE* promotes *EGFR* transcription activity to affect signaling. However, knockdown of epigenetic regulatory factors in HCC cells with low *CRNDE* expression reduced *EGFR* mRNA but not protein expression and further induced cell proliferation. EGFR protein expression may therefore be mediated to maintain normal biological function by other feedback mechanisms under conditions of low expression of *CRNDE* in HCC cells. In a previous study, the long non-coding RNA lnc-*EGFR* was shown to promote human tongue cancer proliferation via upregulation of *EGFR* mRNA and protein, potentially through direct binding [39]. Furthermore, *EGFR*-AS1 and EGFR expression are reported to be positively correlated, with *EGFR*-AS1 promoting the expression of EGFR by inhibiting degradation of its mRNA via direct interactions [40]. Long intergenic non-protein coding RNA 52 (LINC00052) promotes head and neck squamous cell carcinoma (HNSCC) progression through the regulation of EGFR by sponging miR-608 [41]. Our results indicate that *CRNDE* could modulate *EGFR* transcription through epigenetic regulators, further influencing tumorigenesis and growth in HCC. Both the GSEA and Oncomine datasets suggested that the EGFR expression was no difference between normal (N) and tumor (T). However, EGFR expression in our 240 paired HCC tumor (T) tissue specimens was markedly higher relative to normal (N), and it was increased at the advanced pathological stage, larger tumors, and virus infection. These outcomes are consistent with *CRNDE*. Thus, *CRNDE* possibly induces *EGFR* transcription under specific risk factors such as the hepatitis C virus (HCV) or hepatitis B virus (HBV)/HCV infection. Multiple lncRNAs regulate gene transcription through the activity as a scaffold that recruits p300 and enhances H3K9Ac [42–44]. p300 also acts as a coactivator that can interact directly or indirectly with multiple transcription factors to regulate downstream target genes [27, 33]. YY1 is a common multifunctional transcription factor that interacts with p300. In glioblastoma, YY1 acts as an oncogene that stimulates EGFR expression via miRNA effects, in turn, promoting cellular proliferation [45]. In this study, we showed that *CRNDE* regulates p300/YY1 interactions and promotes the binding of the complex to the *EGFR* promoter in association with H3K9Ac, in turn, influencing *EGFR* transcriptional activity.

In contrast to genetic mutations, epigenetic alterations are reversible. Given the importance of epigenetic marks in tumorigenesis, the identification of corresponding inhibitors has attracted extensive attention [36]. Considerable evidence has emerged showing that C646 influences tumor proliferation, metastasis, and drug resistance through suppression of lncRNA transcription owing to its function as a p300 inhibitor [46, 47]. C646 is under investigation as a promising candidate anti-tumor drug [48], but the specific mechanisms underlying its inhibitory effect on sorafenib resistance in HCC remain largely unclear. Data from the current study confirmed that tumor growth and sorafenib resistance in HCC were improved by the epigenetic inhibitor. Under conditions of high *CRNDE* expression, C646 treatment reduced chromatin relaxation, in turn, decreasing YY1 binding to the *EGFR* promoter and suppressing transcriptional activity, resulting in a significant decrease in sorafenib resistance and prolonged overall survival. Additionally, we also found no significant regulation of *EGFR* mRNA and protein levels by sorafenib treatment. These results are consistent with The phase 3 STORM trial, which compared sorafenib with placebo and showed that *EGFR* is not significantly different between the placebo group and the sorafenib-treated group (GSE109211) ([49]). Thus, EGFR played an important mediator in regulating sorafenib resistant phenotype, but its expression level is only repressed by C646 treatment.

In summary, we have uncovered a novel molecular mechanism involving *CRNDE* that underlies HCC proliferation and sorafenib resistance through chromatin relaxation and induction of epigenetic regulatory complex binding to the *EGFR* promoter to enhance its transcriptional activity. Our findings support the development of effective therapeutic strategies based on p300 inhibitors that significantly inhibit HCC growth and decrease sorafenib resistance to extend total median survival. *CRNDE* overexpression clearly contributes to the development and progression of HCC, supporting its utility as a target for blocking or reversing tumor progression and sorafenib resistance. Overall, p300 inhibitors, such as C646, may present an efficient alternative therapeutic option to combat sorafenib resistance in patients with advanced HCC.

## Supplementary Information

**Additional file 1: Fig. S1.** (**A** and **B**) Data are presented as relative expression levels in tumor tissues. *CRNDE* expression was significantly increased in HCC patients with HCV, HBV+HCV and cirrhosis. Statistical significance (*P*-value) was calculated with the two-tailed Student's t-test for a single comparison between two groups. Data are presented as mean ± SD (*P<0.05; **P<0.01; ***P<0.001).

**Additional file 2: Fig. S2.** (**A** and **B**) Hep3B and SkHep1 cells were infected with shRNAs of *CRNDE* (shCRNDE#1 and shCRNDE#2) and cell numbers assessed relative to control cell groups via the proliferation assay. (**C** and **D**) Huh7 and J7 cells were transfected with pcDNA3-control or pcDNA3-*CRNDE*. Cell numbers were assessed after 48 h using the proliferation assay and expressed relative to control cells. (**E**) Nude mice were subcutaneously injected with the two groups (pcDNA3-control and *CRNDE* overexpression, N=11 per group) of J7 cells. Representative tumors were excised after 21 days. Sizes (cm) are indicated at the bottom. (**F**) Examination of cell line-derived xenograft tumor sizes of J7 cells expressing pcDNA3-control or *CRNDE* overexpression plasmids in nude mice. (**G**) Survival rates of nude mice injected with J7 cells containing pcDNA3-control or *CRNDE* overexpression plasmid. Kaplan–Meier plots were generated, using standard tumor sizes (200 mm^3^) as a criterion for euthanasia. (**H** and **I**) Hep3B and SkHep1 cell matrigel migration and invasion of control (shLuc#1 and shLuc#2) or *CRNDE* depletion (shCRNDE#1 and shCRNDE#2) groups were determined using the transwell assay. Scale bar = 200 μm. (**J** and **K**) Huh7 and J7 cell matrigel migration and invasion abilities of pcDNA3-control or *CRNDE* overexpression groups were determined using the transwell assay. Scale bar = 200 μm. (**L** and **M**) Hep3B and SkHep1 were quantified based on transwell assay data (**H** and **I**). (**N** and **O**) Huh7 and J7 cells were quantified based on transwell assay data (**J** and **K**). (**P**) Control or *CRNDE*-depleted Hep3B tumor cells were injected into the tail vein of SCID mice. Representative macroscopic images of liver metastases in formalin-fixed organs. Sizes (cm) are indicated at the bottom. (**Q**) Huh7 tumor cells containing pcDNA3-control or *CRNDE* overexpression plasmids were injected into the tail veins of SCID mice. Representative macroscopic images of liver metastases in formalin-fixed organs. Sizes (cm) are indicated at the bottom. (**R** and **S**) Hep3B and SkHep1 cells from control and *CRNDE*-depletion groups were incubated with or without sorafenib and cell viability relative to the control cells assessed after 72 h using the MTT assay. (**T** and **U**) Huh7 and J7 cells from pcDNA3-control and *CRNDE* overexpression groups were incubated with or without sorafenib and cell viability relative to control cells assessed after 72 h using the MTT assay. Statistical significance (*P* value) was calculated with the two-tailed Student's t-test for a single comparison between two groups. Data are presented as mean ± SD (*p<0.05; **p<0.01; ***p<0.001; ****p<0.0001).

**Additional file 3: Fig. S3.** (**A**) p-EGFR, EGFR, p-STAT3, and STAT3 protein levels in SkHep1 cells from control and *CRNDE* depletion groups were determined by western blot. (**B**) p-EGFR, EGFR, p-STAT3, and STAT3 protein levels in J7 cells from pcDNA3 and *CRNDE* overexpression groups were determined by western blot. (**C**) *EGFR* mRNA expression in SkHep1of control and *CRNDE* depletion groups were determined by qRT-PCR. (**D**) *EGFR* mRNA expression in J7 of pcDNA3 and *CRNDE* overexpression were determined by qRT-PCR. (**E**–**H**) qRT-PCR analysis of the efficiency of *CRNDE* silencing in Hep3B and SkHep1 cells and *CRNDE* overexpression in Huh7 and J7 cells. (**I**) IF analysis of EGFR protein expression in Huh7 cells (pcDNA3-control and *CRNDE* overexpression).

**Additional file 4: Fig. S3.** (**J**) The *EGFR* mRNA expression level in human HCC (T) (n=369) compared to adjacent normal tissue (N) (n=160) were no different, as calculated from The Cancer Genome Atlas datasets. (**K**) There are no different expressions of EGFR in HCC (n=35) compared with liver (n=10) specimens from the Wurmbach Liver dataset in the Oncomine database. (**L**–**N**) Data are presented as relative expression levels in tumor tissues. *EGFR* expression was significantly increased in 240 paired in-house HCC patients with higher pathological stages, larger tumors, and HBC, HCV, HBV+HCV infection. (**O**) J7 cells were transfected with pcDNA3-control or pcDNA3-CRNDE plasmids with siRNA-induced silencing of EGFR. Cell numbers were assessed after 48 h using the proliferation assay and expressed relative to control cells. (**P**) J7 cells from pcDNA3-control and CRNDE-overexpression with siRNA-induced silencing of EGFR were incubated with or without sorafenib and cell viability relative to control cells was assessed after 72 h using the MTT assay. (**Q**). EGFR protein levels in J7 cells from pcDNA3-control and CRNDE-overexpression with siRNA-induced silencing of EGFR were determined by western blot. Statistical significance (*P*-value) was calculated with the two-tailed Student's t-test for a single comparison between two groups. Data are presented as mean ± SD (*P<0.05; **P<0.01; ***P<0.001).

**Additional file 5: Fig. S4.** (**A**) Relative luciferase activities of each promoter were determined in SkHep1 with conditions of CRNDE depletion cells by reporter assay, taking control (shLuc) EGFR promoter as 100%. (**B**) Relative luciferase activities of each promoter were determined in J7 with conditions of CRNDE overexpression cells by reporter assay, taking control (pcDNA3) EGFR promoter as 100%. (**C** and **D**) Subcellular fractionation of *CRNDE* in Hep3B and SkHep1 cells. qRT-PCR analysis using GAPDH and U1 as cytoplasmic and nuclear localization markers revealed that *CRNDE* is primarily located in the nucleus. (**E**) H3K9Ac, H3K27Ac, and Histone H3 protein levels in SkHep1 cells from control and *CRNDE* depletion groups were determined by western blot. (**G**) H3K9Ac, H3K27Ac, and Histone H3 protein levels in J7 cells from pcDNA3-control and *CRNDE*-overexpression groups were determined by western blot. (**G**) Relative luciferase activity of EGFR promoter in J7 cells transfected with *CRNDE*-overexpression plasmid and using shRNA for silencing of p300 were determined by reporter assay. (**H**) J7 in pcDNA3-control and *CRNDE*-overexpression groups were incubated with DMSO or 20 μM C646 and EGFR, H3K9Ac, H3K27Ac, and histone H3 protein levels were assessed after 48 h by western blot. (**I**) And EGFR mRNA level was assessed by qRT-PCR. (**J**) J7 in pcDNA3-control and CRNDE-overexpression groups were incubated with DMSO or 20 μM C646 and relative luciferase activities of the promoter were assessed after 48 h by reporter assay. Statistical significance (*P*-value) was calculated with the two-tailed Student's t-test for a single comparison between two groups. Data are presented as mean ± SD (*P<0.05; **P<0.01; ***P<0.001).

**Additional file 6: Fig. S5**. (**A** and **B**) YY1 and EGFR protein and *EGFR* mRNA expression in J7 cells under conditions of *CRNDE* overexpression and silencing of YY1 using shRNA were determined by western blot and qRT-PCR analysis, respectively. (**C**) Relative luciferase activity of *EGFR* promoter in J7 cells transfected with *CRNDE* overexpression plasmid and using shRNA for silencing of YY1 were determined by reporter assay. (**D**) Reporter assay of relative luciferase activity of each promoter in J7 cells transfected with pcDNA3-control and *CRNDE* overexpression constructs. Luciferase activities of *EGFR* promoter with mutant YY1 binding site 1 (M1), YY1 binding site 2 (M2), and YY1 binding site 1 and 2 (M3), taking wild-type (WT) *EGFR* promoter activity as 100%. (**E** and **F**) ChIP assay of YY1 on the *EGFR* promoter in Hep3B transfected with control and *CRNDE*-depletion constructs and in Huh7 cells transfected with pcDNA3-control and *CRNDE* overexpression plasmids. Co-IP DNA for negative control region (*GAPDH*) was detected via q-PCR. (**G** and **H**) YY1 binding to the *EGFR* promoter in SkHep1 cells transfected with control or *CRNDE*-depletion constructs and in J7 cells transfected with pcDNA3-control and *CRNDE* overexpression plasmids were determined by ChIP assay. Images of representative gels of ChIP PCR products. Chromatin of cells subjected to the indicated treatments was immunoprecipitated with YY1 antibody or IgG control. Co-IP DNA was detected with PCR for the YY1 binding region (*EGFR*) or negative control region (*GAPDH*). Statistical significance (*P* value) was calculated with the two-tailed Student's t-test for a single comparison between two groups. Data are presented as mean ± SD (*p<0.05; **p<0.01; ***p<0.001).

**Additional file 7: Fig. S6**. (**A** and **B**) SkHep1 and J7 extracts were immunoprecipitated using mouse IgG or p300 antibody. Immunoprecipitation of p300-associated RNA was reverse transcription and quantitative PCR were performed and presented as relative fold of RNA enrichment. *GAPDH* was used as a negative control. (**C** and **F**) p300/YY1 complex binding to the *EGFR* promoter in Hep3B transfected with control and *CRNDE*-depletion constructs and in Huh7 cells transfected with pcDNA3-control and *CRNDE* overexpression plasmids were determined by re-ChIP assay. (**D** and **E**) re-ChIP assay of p300/YY1 complex binding to the *EGFR* promoter in Hep3B and SkHep1 transfected with control and *CRNDE*-depletion constructs. (**G** and **H**) re-ChIP assay of p300/YY1 complex binding to the *EGFR* promoter in Huh7 and J7 transfected with control and *CRNDE* overexpression constructs. (**I** and **L**) H3K9Ac on *EGFR* promoter in SkHep1 transfected with control and *CRNDE*-depletion constructs and in J7 cells transfected with pcDNA3-control and *CRNDE* overexpression plasmids were determined by ChIP assay. (**J** and **K**) ChIP assay of H3K9Ac on the *EGFR* promoter in Hep3B and SkHep1 transfected with control and *CRNDE*-depletion constructs. (**M** and **N**) ChIP assay of H3K9Ac on the *EGFR* promoter in Huh7 and J7 transfected with control and *CRNDE* overexpression constructs. (**O** and **R**) H3K9Ac/YY1 complex binding to *EGFR* promoter in SkHep1 transfected with control and *CRNDE*-depletion constructs and in J7 cells transfected with pcDNA3-control and *CRNDE* overexpression plasmids were determined by re-ChIP assay. (**P** and **Q**) re-ChIP assay of H3K9Ac/YY1 complex binding to the *EGFR* promoter in Hep3B and SkHep1 transfected with control and *CRNDE*-depletion constructs. (**S** and **T**) re-ChIP assay of H3K9Ac/YY1 complex binding to the *EGFR* promoter in Huh7 and J7 transfected with control and *CRNDE* overexpression constructs. (**U**) H3K9Ac binding to *EGFR* promoter in Huh7 cells with *CRNDE* overexpression and incubation with or without C646 treatment were determined by ChIP assay. (**V**) H3K9Ac/YY1 binding to *EGFR* promoter in Huh7 cells with *CRNDE* overexpression and incubation with or without C646 treatment were determined by re-ChIP assay. Statistical significance (*P* value) was calculated with the two-tailed Student's t-test for a single comparison between two groups. Data are presented as mean ± SD (*p<0.05; **p<0.01; ***p<0.001).

**Additional file 8: Fig. S7.** (**A** and **B**) Matrigel migration and invasion of pcDNA3-control or *CRNDE* overexpression Huh7 cell groups with shRNA-mediated silencing of p300 (shp300#1 and shp300#2) and YY1 (shYY1#1 and shYY1#2), determined via transwell assay. Scale bar = 200 μm. (**C** and **D**) Huh7 cells were quantified based on transwell assay data (**A** and **B**). Statistical significance (*P* value) was calculated with the two-tailed Student's t-test for a single comparison between two groups. Data are presented as mean ± SD (*p<0.05; **p<0.01; ***p<0.001).

**Additional file 9: Fig. S8**. (**A**) J7 cells from pcDNA3-control and *CRNDE* overexpression groups were incubated with or without sorafenib and cell viability relative to control cells assessed after 72 h using the MTT assay. Statistical significance (*P* value) was calculated with the two-tailed Student's t-test for a single comparison between two groups. Data are presented as mean ± SD (*p<0.05; **p<0.01; ***p<0.001).

**Additional file 10.** The shRNA and siRNA oligonucleotides sequences.

**Additional file 11.** The quantitative reverse transcription-PCR (qRT-PCR) primer sequences.

**Additional file 12.** The Chromatin immunoprecipitation (ChIP) and re-ChIP primer sequences.

**Additional file 13.** The primers used for construction of EGFR promoter mutant fragments.

**Additional file 14.** The western blot and Immunohistochemistry primary antibodies against.

## Data Availability

All other data are within the main text or its Supplementary Information.
