# Supplementary figures and images for: CRNDE acts as an epigenetic modulator of the p300/YY1 complex to promote HCC progression and therapeutic resistance
Source: Clin Epigenetics. 2022 Aug 23;14:106. doi: 10.1186/s13148-022-01326-3 (PMC9400329; doi:10.1186/s13148-022-01326-3)

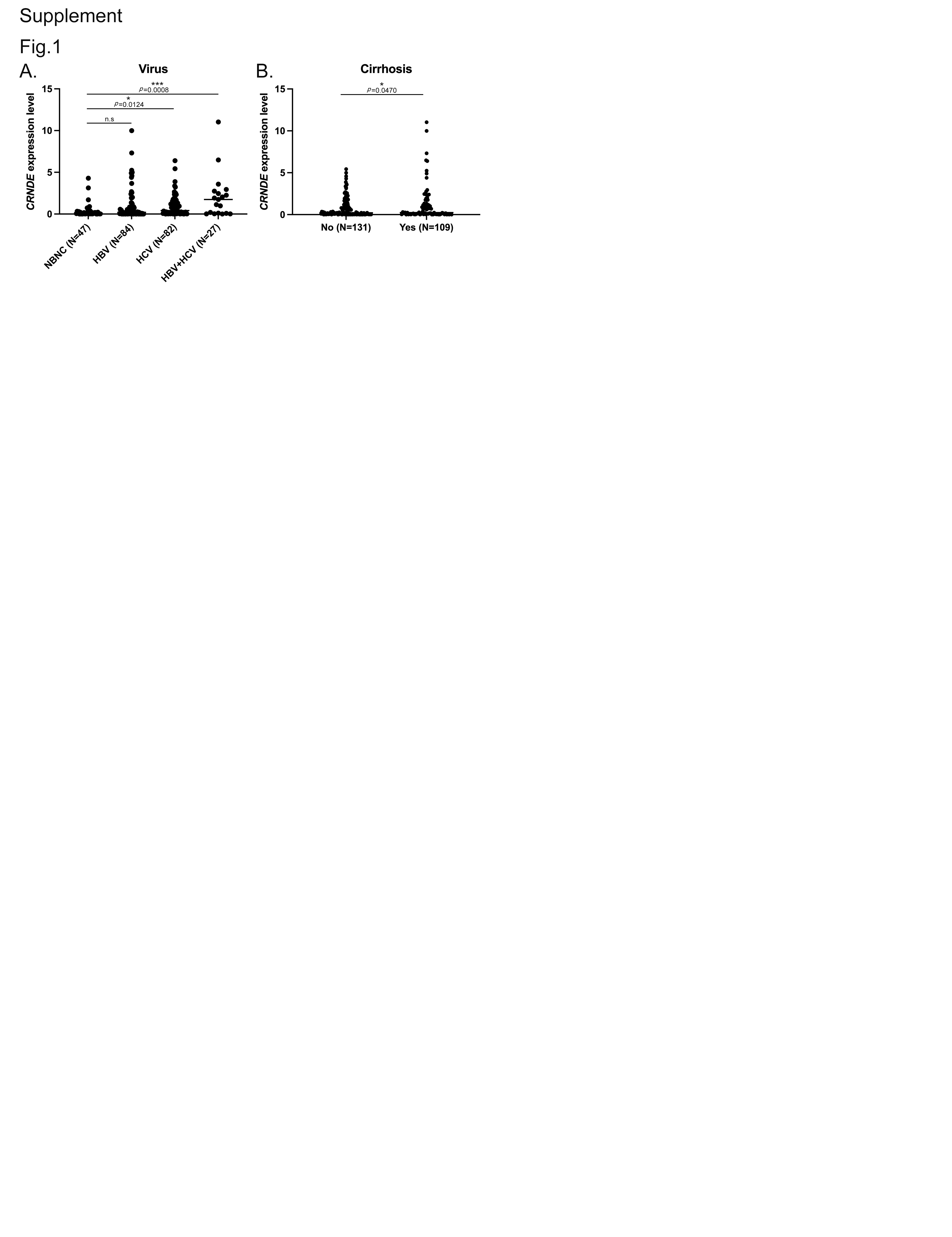

Supplement: Supplementary file 1 — Additional file 1: Fig. S1. (A and B) Data are presented as relative expression levels in tumor tissues. CRNDE expression was significantly increased in HCC patients with HCV, HBV+HCV and cirrhosis. Statistical significance (P-value) was calculated with the two-tailed Student's t-test for a single comparison between two groups. Data are presented as mean ± SD (*P<0.05; **P<0.01; ***P<0.001). [file 13148_2022_1326_MOESM1_ESM.tif]

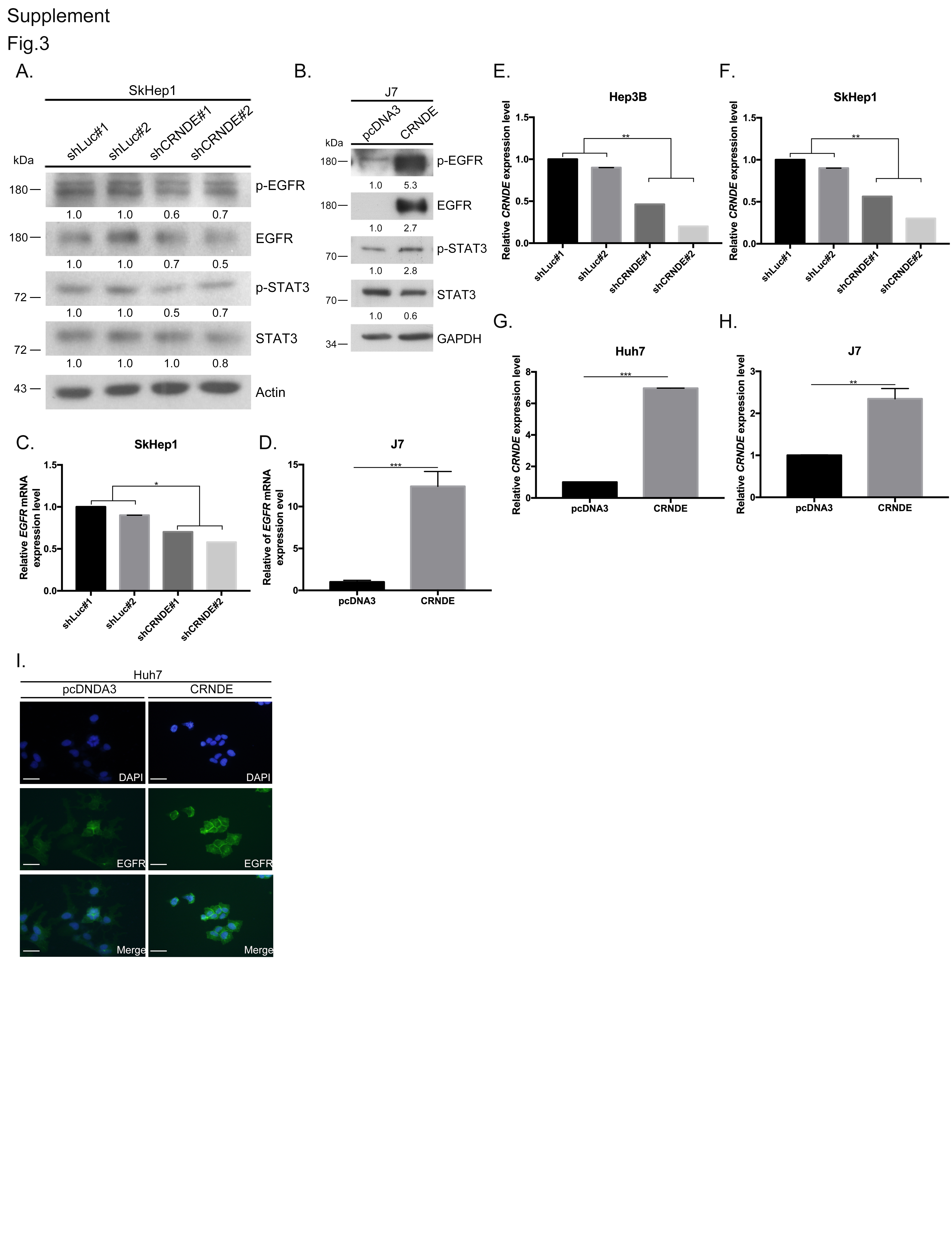

Supplement: Supplementary file 3 — Additional file 3: Fig. S3. (A) p-EGFR, EGFR, p-STAT3, and STAT3 protein levels in SkHep1 cells from control and CRNDE depletion groups were determined by western blot. (B) p-EGFR, EGFR, p-STAT3, and STAT3 protein levels in J7 cells from pcDNA3 and CRNDE overexpression groups were determined by western blot. (C) EGFR mRNA expression in SkHep1of control and CRNDE depletion groups were determined by qRT-PCR. (D) EGFR mRNA expression in J7 of pcDNA3 and CRNDE overexpression were determined by qRT-PCR. (E–H) qRT-PCR analysis of the efficiency of CRNDE silencing in Hep3B and SkHep1 cells and CRNDE overexpression in Huh7 and J7 cells. (I) IF analysis of EGFR protein expression in Huh7 cells (pcDNA3-control and CRNDE overexpression). [file 13148_2022_1326_MOESM3_ESM.tif]

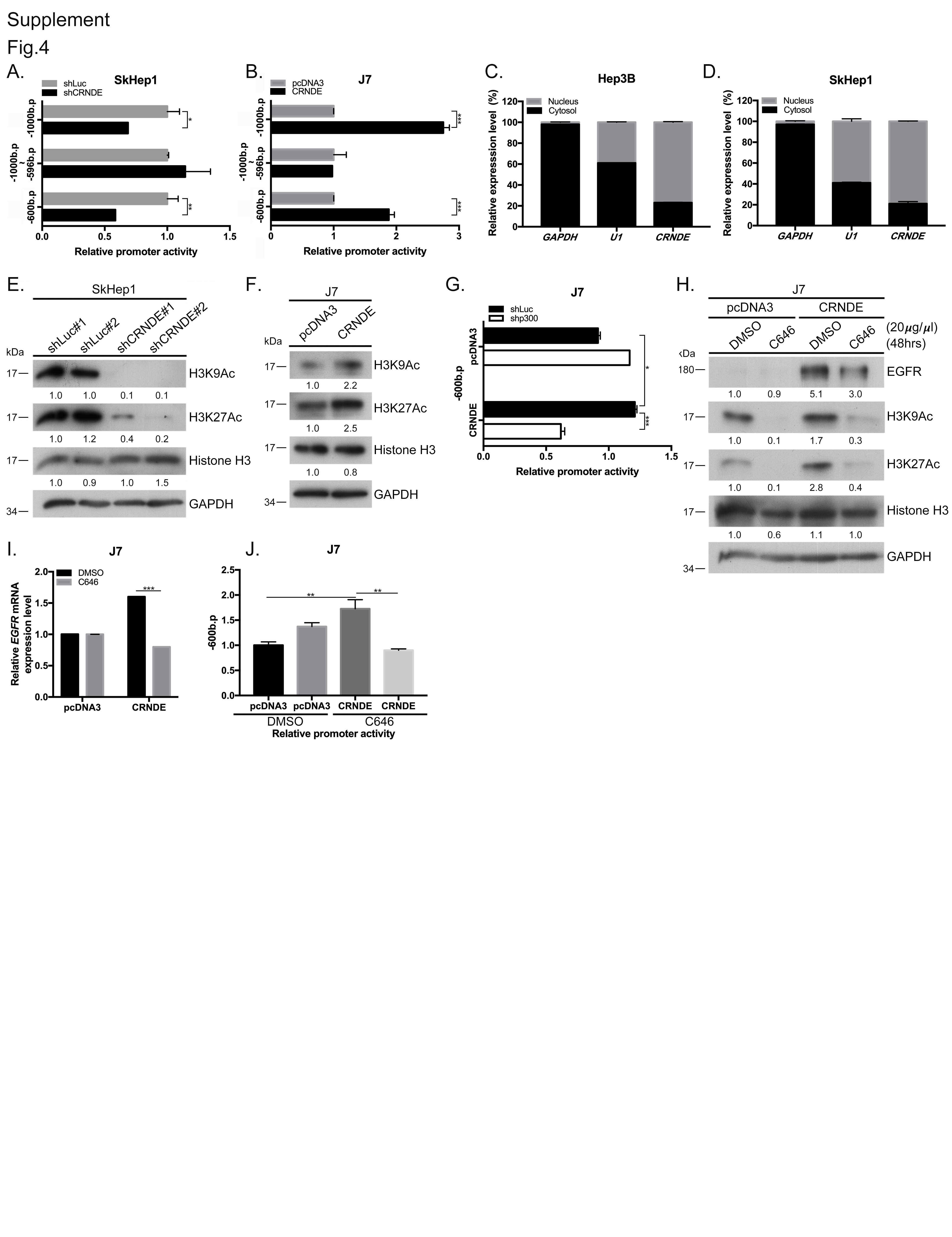

Supplement: Supplementary file 5 — Additional file 5: Fig. S4. (A) Relative luciferase activities of each promoter were determined in SkHep1 with conditions of CRNDE depletion cells by reporter assay, taking control (shLuc) EGFR promoter as 100%. (B) Relative luciferase activities of each promoter were determined in J7 with conditions of CRNDE overexpression cells by reporter assay, taking control (pcDNA3) EGFR promoter as 100%. (C and D) Subcellular fractionation of CRNDE in Hep3B and SkHep1 cells. qRT-PCR analysis using GAPDH and U1 as cytoplasmic and nuclear localization markers revealed that CRNDE is primarily located in the nucleus. (E) H3K9Ac, H3K27Ac, and Histone H3 protein levels in SkHep1 cells from control and CRNDE depletion groups were determined by western blot. (G) H3K9Ac, H3K27Ac, and Histone H3 protein levels in J7 cells from pcDNA3-control and CRNDE-overexpression groups were determined by western blot. (G) Relative luciferase activity of EGFR promoter in J7 cells transfected with CRNDE-overexpression plasmid and using shRNA for silencing of p300 were determined by reporter assay. (H) J7 in pcDNA3-control and CRNDE-overexpression groups were incubated with DMSO or 20 μM C646 and EGFR, H3K9Ac, H3K27Ac, and histone H3 protein levels were assessed after 48 h by western blot. (I) And EGFR mRNA level was assessed by qRT-PCR. (J) J7 in pcDNA3-control and CRNDE-overexpression groups were incubated with DMSO or 20 μM C646 and relative luciferase activities of the promoter were assessed after 48 h by reporter assay. Statistical significance (P-value) was calculated with the two-tailed Student's t-test for a single comparison between two groups. Data are presented as mean ± SD (*P<0.05; **P<0.01; ***P<0.001). [file 13148_2022_1326_MOESM5_ESM.tif]

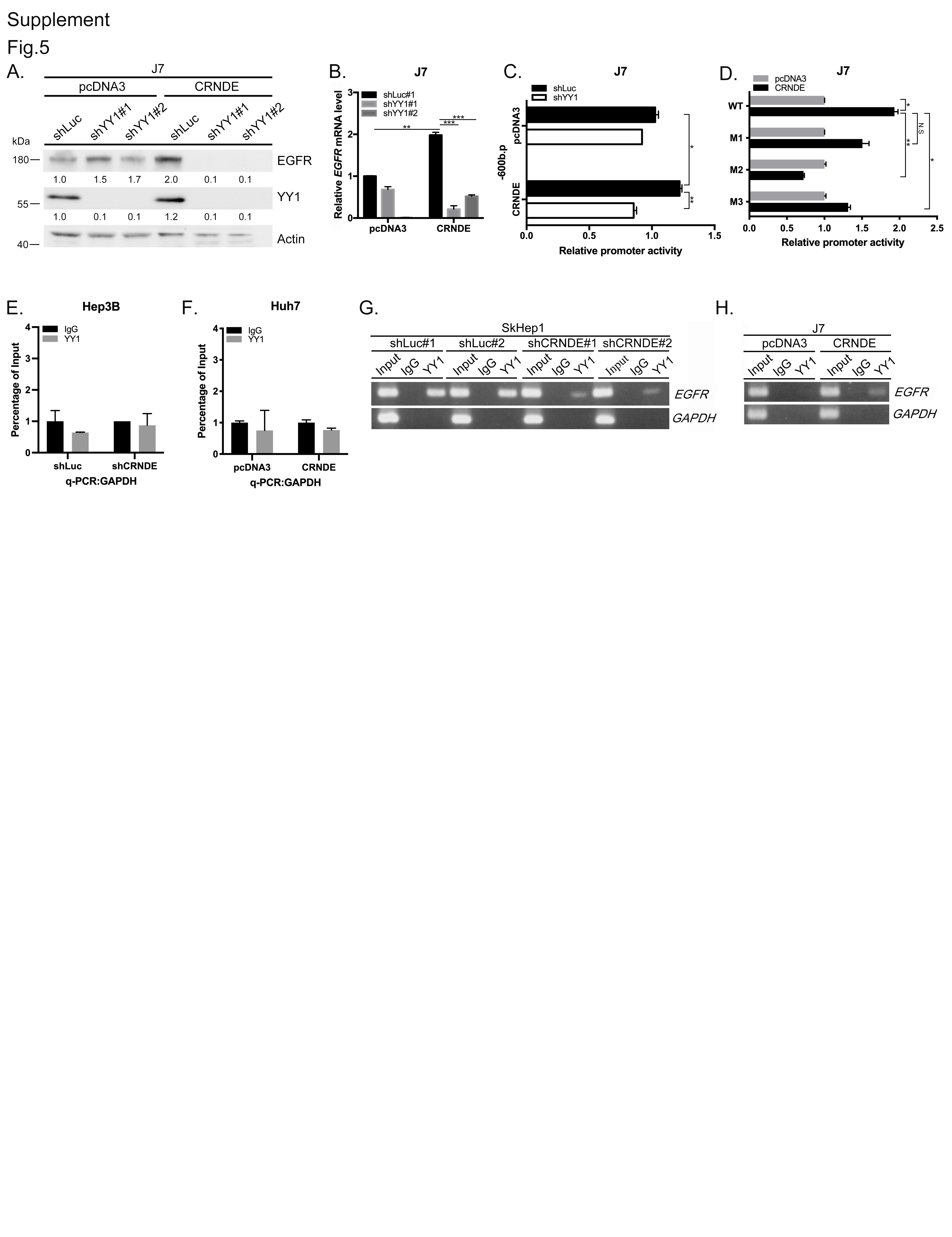

Supplement: Supplementary file 6 — Additional file 6: Fig. S5. (A and B) YY1 and EGFR protein and EGFR mRNA expression in J7 cells under conditions of CRNDE overexpression and silencing of YY1 using shRNA were determined by western blot and qRT-PCR analysis, respectively. (C) Relative luciferase activity of EGFR promoter in J7 cells transfected with CRNDE overexpression plasmid and using shRNA for silencing of YY1 were determined by reporter assay. (D) Reporter assay of relative luciferase activity of each promoter in J7 cells transfected with pcDNA3-control and CRNDE overexpression constructs. Luciferase activities of EGFR promoter with mutant YY1 binding site 1 (M1), YY1 binding site 2 (M2), and YY1 binding site 1 and 2 (M3), taking wild-type (WT) EGFR promoter activity as 100%. (E and F) ChIP assay of YY1 on the EGFR promoter in Hep3B transfected with control and CRNDE-depletion constructs and in Huh7 cells transfected with pcDNA3-control and CRNDE overexpression plasmids. Co-IP DNA for negative control region (GAPDH) was detected via q-PCR. (G and H) YY1 binding to the EGFR promoter in SkHep1 cells transfected with control or CRNDE-depletion constructs and in J7 cells transfected with pcDNA3-control and CRNDE overexpression plasmids were determined by ChIP assay. Images of representative gels of ChIP PCR products. Chromatin of cells subjected to the indicated treatments was immunoprecipitated with YY1 antibody or IgG control. Co-IP DNA was detected with PCR for the YY1 binding region (EGFR) or negative control region (GAPDH). Statistical significance (P value) was calculated with the two-tailed Student's t-test for a single comparison between two groups. Data are presented as mean ± SD (*p<0.05; **p<0.01; ***p<0.001). [file 13148_2022_1326_MOESM6_ESM.tif]

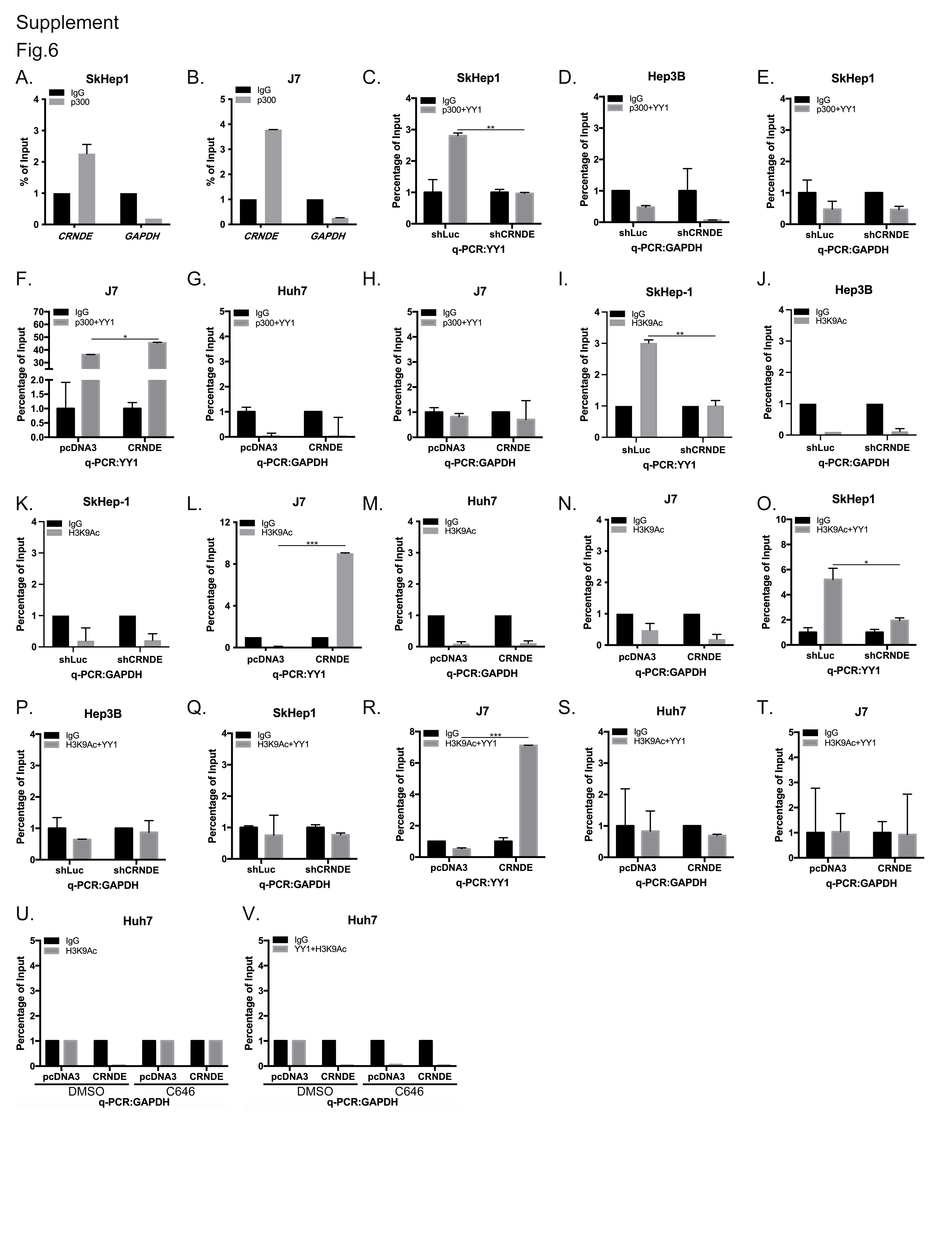

Supplement: Supplementary file 7 — Additional file 7: Fig. S6. (A and B) SkHep1 and J7 extracts were immunoprecipitated using mouse IgG or p300 antibody. Immunoprecipitation of p300-associated RNA was reverse transcription and quantitative PCR were performed and presented as relative fold of RNA enrichment. GAPDH was used as a negative control. (C and F) p300/YY1 complex binding to the EGFR promoter in Hep3B transfected with control and CRNDE-depletion constructs and in Huh7 cells transfected with pcDNA3-control and CRNDE overexpression plasmids were determined by re-ChIP assay. (D and E) re-ChIP assay of p300/YY1 complex binding to the EGFR promoter in Hep3B and SkHep1 transfected with control and CRNDE-depletion constructs. (G and H) re-ChIP assay of p300/YY1 complex binding to the EGFR promoter in Huh7 and J7 transfected with control and CRNDE overexpression constructs. (I and L) H3K9Ac on EGFR promoter in SkHep1 transfected with control and CRNDE-depletion constructs and in J7 cells transfected with pcDNA3-control and CRNDE overexpression plasmids were determined by ChIP assay. (J and K) ChIP assay of H3K9Ac on the EGFR promoter in Hep3B and SkHep1 transfected with control and CRNDE-depletion constructs. (M and N) ChIP assay of H3K9Ac on the EGFR promoter in Huh7 and J7 transfected with control and CRNDE overexpression constructs. (O and R) H3K9Ac/YY1 complex binding to EGFR promoter in SkHep1 transfected with control and CRNDE-depletion constructs and in J7 cells transfected with pcDNA3-control and CRNDE overexpression plasmids were determined by re-ChIP assay. (P and Q) re-ChIP assay of H3K9Ac/YY1 complex binding to the EGFR promoter in Hep3B and SkHep1 transfected with control and CRNDE-depletion constructs. (S and T) re-ChIP assay of H3K9Ac/YY1 complex binding to the EGFR promoter in Huh7 and J7 transfected with control and CRNDE overexpression constructs. (U) H3K9Ac binding to EGFR promoter in Huh7 cells with CRNDE overexpression and incubation with or without C646 treatment were determined [file 13148_2022_1326_MOESM7_ESM.tif]

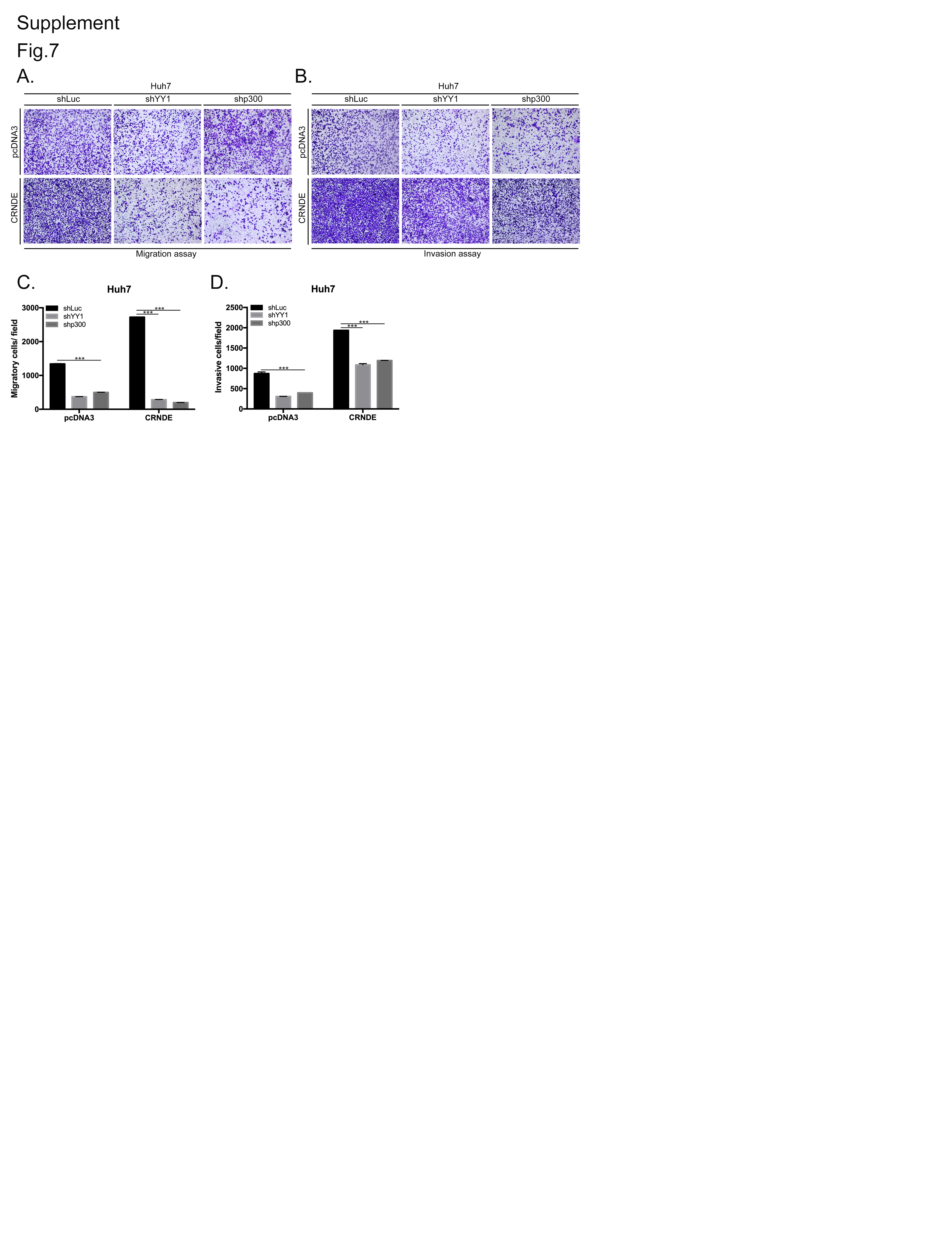

Supplement: Supplementary file 8 — Additional file 8: Fig. S7. (A and B) Matrigel migration and invasion of pcDNA3-control or CRNDE overexpression Huh7 cell groups with shRNA-mediated silencing of p300 (shp300#1 and shp300#2) and YY1 (shYY1#1 and shYY1#2), determined via transwell assay. Scale bar = 200 μm. (C and D) Huh7 cells were quantified based on transwell assay data (A and B). Statistical significance (P value) was calculated with the two-tailed Student's t-test for a single comparison between two groups. Data are presented as mean ± SD (*p<0.05; **p<0.01; ***p<0.001). [file 13148_2022_1326_MOESM8_ESM.tif]

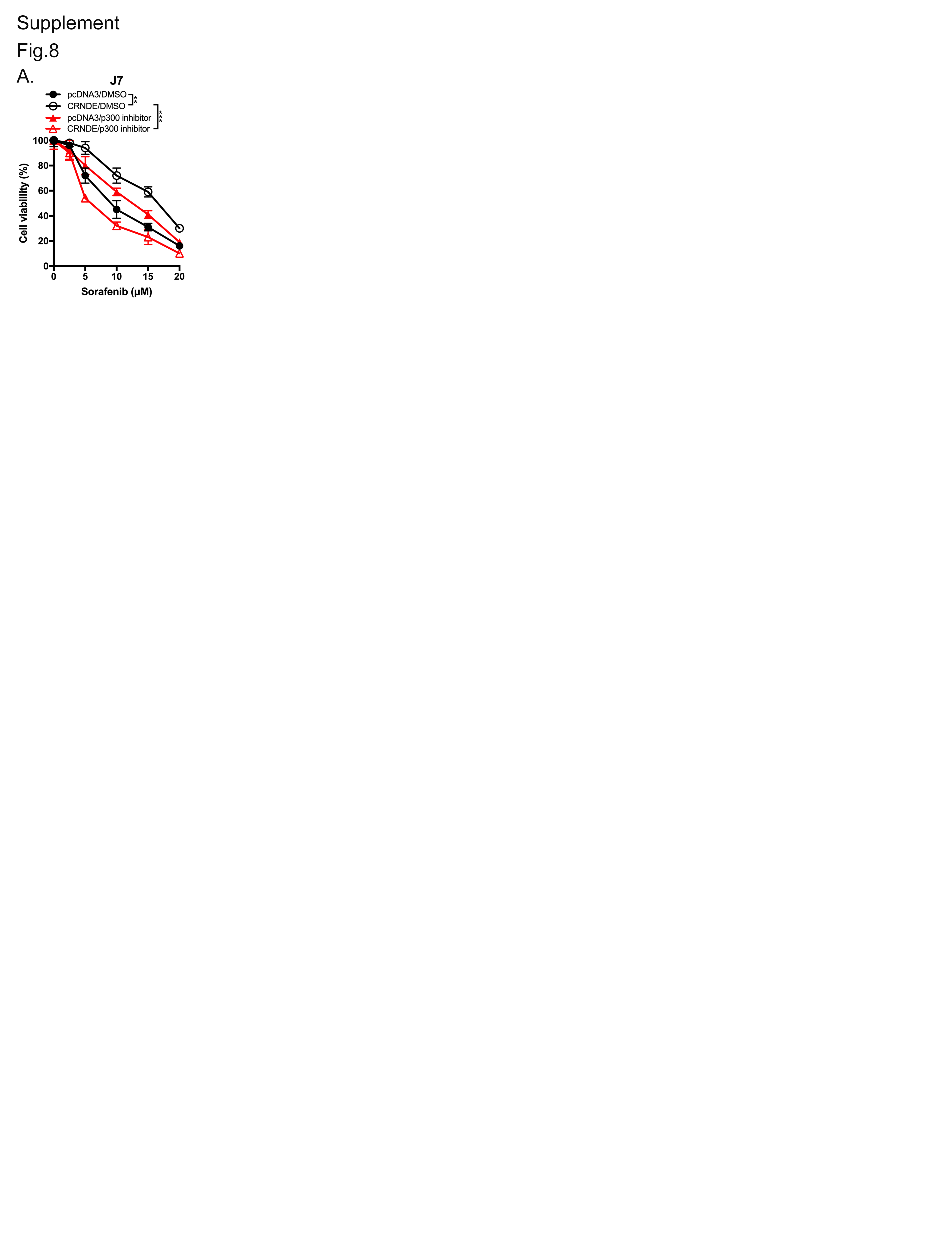

Supplement: Supplementary file 9 — Additional file 9: Fig. S8. (A) J7 cells from pcDNA3-control and CRNDE overexpression groups were incubated with or without sorafenib and cell viability relative to control cells assessed after 72 h using the MTT assay. Statistical significance (P value) was calculated with the two-tailed Student's t-test for a single comparison between two groups. Data are presented as mean ± SD (*p<0.05; **p<0.01; ***p<0.001). [file 13148_2022_1326_MOESM9_ESM.tif]
