# Supplementary material for: CRNDE acts as an epigenetic modulator of the p300/YY1 complex to promote HCC progression and therapeutic resistance
Source: Clin Epigenetics. 2022 Aug 23;14:106. doi: 10.1186/s13148-022-01326-3 (PMC9400329; doi:10.1186/s13148-022-01326-3)
Supplement: Supplementary file 10 — Additional file 10. The shRNA and siRNA oligonucleotides sequences. [file 13148_2022_1326_MOESM10_ESM.docx]

Supplement Table 1. The following oligonucleotides were used to construct the shCRNDE#1 and shCRNDE#2, and the plasmid expressing of shRNA list were purchased from the National RNAi Core Facility, and siRNA sequence were purchased from the Dharmacon (Thermo Fisher Scientific, Lafayette, CO were presented

| shCRNDE#1 | Forward primer | 5’CCGGCTCCAAATGTTGGCTGAAATTCTCGAGAATTTCAGCCAACATTTGGAGTTTTT3' |  |
| --- | --- | --- | --- |
|  | Reverse primer | 5’AATTAAAAACTCCAAATGTTGGCTGAAATTCTCGAGAATTTC AGCCAACATTTGGAG3’ |  |
| shCRNDE#2 | Forward primer | 5’CCGGAGTTCTCTTGTAGGATGCCACCTCGAGGTGGCATCCTACAAGAGAACTTTTTT3’ |  |
|  | Reverse primer | 5’AATTAAAAAAGTTCTCTTGTAGGATGCCACCTCGAGGTGGCATCCTACAAGAGAACT3' |  |
| luciferase shRNA (negative control) |  |  | TRCN0000072244 |
| P300 shRNA (shp300#1) |  |  | TRCN0000009882 |
| P300 shRNA (shp300#2) |  |  | TRCN0000009884 |
| YY1 shRNA (shpYY1#1) |  |  | TRCN0000019894 |
| YY1 shRNA (shpYY1#2) |  |  | TRCN0000019898 |
| siEGFR |  | CAAAGUGUGUAACGGAAUA;  CCAUAAAUGCUACGAAUAU;  GUAACAAGCUCACGCAGUU;  CAGAGGAUGUUCAAUAACU |  |
