# Supplementary material for: CRNDE acts as an epigenetic modulator of the p300/YY1 complex to promote HCC progression and therapeutic resistance
Source: Clin Epigenetics. 2022 Aug 23;14:106. doi: 10.1186/s13148-022-01326-3 (PMC9400329; doi:10.1186/s13148-022-01326-3)
Supplement: Supplementary file 11 — Additional file 11. The quantitative reverse transcription-PCR (qRT-PCR) primer sequences. [file 13148_2022_1326_MOESM11_ESM.docx]

Supplement Table 2. The quantitative reverse transcription-PCR (qRT- PCR) primer sequences (200 nM) are as follows.

| *CRNDE* | NR_034106.3 | Forward primer | 5’-AAATCAAAGTGCTCGAGTGGTTT-3’ |
| --- | --- | --- | --- |
|  |  | Reverse primer | 5’-CCTTCTTCTGCGTGACAACTGA-3’ |
| *EGFR* | NM_005228 | Forward primer | 5’-CGGGACATAGTCAGCAGTG-3’ |
|  |  | Reverse primer | 5’-GCTGGGCACAGATGATTTTG-3’ |
| *EP300* | NM_001429 | Forward primer | 5’-CAGATTGATCCCAGCTCCAT-3’ |
|  |  | Reverse primer | 5’-GAAAGAAGACTCGGCGTTTG-3’ |
| *YY1* | NM_003403 | Forward primer | 5’- CAGGCCAGGTTGGTCATAGG-3’ |
|  |  | Reverse primer | 5’-CCTGTCAGACAAGCCAAGGT-3’ |
| *GAPDH* | NM 000996 | Forward primer | 5’- TCCTGCACCACCAACTGCTT-3’ |
|  |  | Reverse primer | 5’-GAGGGGGCCATCCACGTCTT-3’ |
| *18S* |  | Forward primer | 5’- CGAGCCGCCTGGATACC-3’ |
|  |  | Reverse primer | 5’- CCTCAGTTCCGAAAACCAACAA-3’ |
