# Supplementary material for: CRNDE acts as an epigenetic modulator of the p300/YY1 complex to promote HCC progression and therapeutic resistance
Source: Clin Epigenetics. 2022 Aug 23;14:106. doi: 10.1186/s13148-022-01326-3 (PMC9400329; doi:10.1186/s13148-022-01326-3)
Supplement: Supplementary file 12 — Additional file 12. The Chromatin immunoprecipitation (ChIP) and re-ChIP primer sequences. [file 13148_2022_1326_MOESM12_ESM.docx]

Supplement Table 3. The Chromatin immunoprecipitation (ChIP) and re-ChIP primer sequences (200 nM) are as follows.

| YY1 binding site | Forward primer | 5’-CGGGGACCGGGTCCAGAGG-3’ |
| --- | --- | --- |
|  | Reverse primer | 5’-TTTAGAGAACCATGCGGAGC-3’ |
| GAPDH binding site (negative control) | Forward primer | 5’-TACTAGCGGTTTTACGGGCG-3’ |
|  | Reverse primer | 5’-TCGAACAGGAGCAGAGAGCGA-3' |
