# Supplementary material for: CRNDE acts as an epigenetic modulator of the p300/YY1 complex to promote HCC progression and therapeutic resistance
Source: Clin Epigenetics. 2022 Aug 23;14:106. doi: 10.1186/s13148-022-01326-3 (PMC9400329; doi:10.1186/s13148-022-01326-3)
Supplement: Supplementary file 13 — Additional file 13. The primers used for construction of EGFR promoter mutant fragments. [file 13148_2022_1326_MOESM13_ESM.docx]

Supplement Table 4. The primers used for construction of mutant fragments were as follows.

| YY1 binding site-1 (M1) | Forward primer | 5’AGATTTGGCTCGACCTGGATGTAGGCTGGGCCTGCAAGTC3’ |
| --- | --- | --- |
|  | Reverse primer | 5’GACTTGCAGGCCCAGCCTACATCCAGGTCGAGCCAAATCT3’ |
| YY1 binding site-2 (M2) | Forward primer | 5’GCACCCGCTCCCCTCCACTGCGCCGC CCCACTCCGC3’ |
|  | Reverse primer | 5’GCGGGAGTGGGGCGGCGCATTGGAGGG GAGCGGGTG3’ |
