# Supplementary material for: CRNDE acts as an epigenetic modulator of the p300/YY1 complex to promote HCC progression and therapeutic resistance
Source: Clin Epigenetics. 2022 Aug 23;14:106. doi: 10.1186/s13148-022-01326-3 (PMC9400329; doi:10.1186/s13148-022-01326-3)
Supplement: Supplementary file 14 — Additional file 14. The western blot and Immunohistochemistry primary antibodies against. [file 13148_2022_1326_MOESM14_ESM.docx]

Supplement Table 5. The western blot and Immunohistochemistry primary antibodies against were as follows.

| EGFR | #4267 | Cell Signaling Technology, Beverly, MA, USA |
| --- | --- | --- |
| p-EGFR | #3777 | Cell Signaling Technology, Beverly, MA, USA |
| STAT3 | #4904 | Cell Signaling Technology, Beverly, MA, USA |
| p-STAT3 | #9145 | Cell Signaling Technology, Beverly, MA, USA |
| Myc-Tag | #2278 | Cell Signaling Technology, Beverly, MA, USA |
| Histone H3 | A17562 | ABclonal Inc,, MA, USA |
| p300 | 61401 | Active Motif, Carlsbad, CA, USA |
| H3K9Ac | 61663 | Active Motif, Carlsbad, CA, USA |
| H3K27Ac | 39685 | Active Motif, Carlsbad, CA, USA |
| YY1 | sc-7341 | Santa Cruz Biotechnology, Santa Cruz, CA, USA |
| GAPDH | sc-32233 | Santa Cruz Biotechnology, Santa Cruz, CA, USA |
| EGFR | sc-373746 | Santa Cruz Biotechnology, Santa Cruz, CA, USA |
| Ki-67 | ab16667 | Abcam, Cambridge, MA, USA |
| β-tubulin | MAB3408 | Millipore, Billerica, MA, USA |
